# FliW regulates biofilm formation *in Geobacter sulfurreducens* through interaction with CsrA

**DOI:** 10.1007/s00253-026-13884-0

**Published:** 2026-05-28

**Authors:** Jessica Cholula-Calixto, Guillermo Huerta-Miranda, Bernardo Jaramillo-Rodríguez, Víctor H. Bustamante, Katy Juárez, Alberto Hernández-Eligio

**Affiliations:** 1 Departamento de Microbiología Molecular, Instituto de Biotecnología, Universidad Nacional Autónoma de México, Av. Universidad 2001, Col. Chamilpa CP 62210, Cuernavaca, Morelos, México; 2 Investigador por México, Secretaría de Ciencia, Humanidades, Tecnología e Innovación, Av. Insurgentes Sur 1528, Col. Crédito Constructor, CP 03940, Ciudad de México, México

**Keywords:** CsrA posttranscriptional regulator, Biofilm, *Geobacter sulfurreducens*, Electrochemical, LexA-based genetic system

## Abstract

**Abstract:**

CsrA is a post-transcriptional regulator that controls a wide range of bacterial phenotypes, including carbon metabolism, motility, quorum sensing, virulence, and biofilm formation. In *Geobacter sulfurreducens*, CsrA modulates both biofilm development and extracellular electron transfer in microbial fuel cells. In this study, we further investigated the regulatory mechanism of CsrA and its role in the formation of electroconductive biofilms in *G. sulfurreducens*. Bioelectrochemical analyses revealed that a Δ*csrA* strain produces biofilms with enhanced electroconductivity compared with the wild-type strain. To identify the molecular basis of this regulation, we explore potential CsrA-binding partners, demonstrating that CsrA interacts with the FliW protein, as reported in other bacteria such as *Bacillus subtilis*. By utilizing site-directed mutagenesis, we identified that this interaction requires a conserved asparagine residue (N55) in CsrA, the disruption of which prevents the CsrA-FliW complex formation. Interestingly, *fliW* deletion resulted in reduced biofilm biomass and thickness contrasting with the enhanced phenotypes observed in the Δ*csrA* strain. Furthermore, the Δ*fliW* mutant exhibited a differential expression of transcripts associated with the CsrA regulon in a pattern opposite to that of the Δ*csrA* strain. These findings indicate that FliW antagonizes CsrA activity, and that the N55-mediated interaction is essential for this regulatory control. Collectively, these results allow us to propose a model in which the CsrA–FliW interaction acts as a molecular switch to control biofilm formation in *G. sulfurreducens*. Furthermore, this study expands our understanding of post-transcriptional regulation in electroactive bacteria and highlights the link between regulatory protein interactions, biofilm physiology, and extracellular electron transfer.

**Key points:**

*• FliW regulates CsrA activity, thereby affecting biofilm formation.*

*• CsrA regulation influences electroconductive biofilm development in G. sulfurreducens.*

*• CsrA–FliW regulation offers targets to optimize bioenergy and bioremediation systems.*

**Supplementary Information:**

The online version contains supplementary material available at 10.1007/s00253-026-13884-0.

## Introduction

Bacteria have developed various strategies for responding to changes in their environment and external stimuli. They control the expression of necessary genes at different levels to accomplish this. Post-transcriptional regulation is key because it allows gene expression to be precisely adjusted to the needs of the cells. The post-transcriptional regulator CsrA (carbon storage regulator A) has been extensively studied in bacteria. Overall, the RNA-binding protein CsrA plays a key role in gene regulation and is involved in a wide range of physiological processes, including growth, survival, metabolism, motility, biofilm formation, stress responses, and virulence, at the post-transcriptional level (Gorelik et al. 2024). This protein modulates gene expression by influencing translation initiation and mRNA stability (Potts et al. 2017). CsrA functions as a homodimer that binds to conserved GGA motifs located within the 5′ untranslated regions (5′-UTRs) of target mRNAs. This binding alters RNA secondary structure and often overlaps the Shine–Dalgarno (SD) sequence, thereby interfering with ribosome binding and inhibiting translation initiation (Kulkarni et al. 2006). CsrA is conserved across many bacterial species and has been extensively studied in *Gammaproteobacteria*, including *Escherichia coli*, *Pseudomonas aeruginosa*, *Legionella pneumophila*, *Vibrio cholerae,* and *Salmonella* spp. (Vakulskas et al. 2015).

Two main mechanisms regulating CsrA activity have been described. The first has principally been described in *Gammaproteobacteria* and consists of a competitive mechanism involving noncoding small RNAs (sRNAs). These sRNAs antagonize with CsrA activity by binding directly to the protein, thus preventing interaction with target mRNAs (Vakulskas et al. 2015). The second has been described in some species of the classes *Bacilli* (*Bacillus subtilis* and *Geobacillus thermodenitrificans*), *Epsilonproteobacteria* (*Campylobacter jejuni*), and *Clostridia* (*Clostridioides difficile*), involving an allosteric mechanism (Mukherjee et al. 2016; Altagoer et al. 2016; Bogacz et al. 2021; Oshiro et al. 2021; Zhu et al. 2025). This mechanism is mediated by the FliW protein, which binds to CsrA in a noncompetitive manner. FliW exhibits high specificity for CsrA and forms a heterotetrameric complex. In *Bacillus subtilis*, FliW also interacts with flagellin (Hag), whereas in *Clostridioides difficile*, it is regulated by FliC (Mukherjee et al. 2016; Altagoer et al. 2016; Zhu et al. 2025). The *csrA* and *fliW* genes are conserved in the bacterial genomes of other classes, such as *Thermotogae* (*Thermotoga maritima*) and *Desulfuromonadia* (*Geobacter sulfurreducens*); however, their function has not been studied to date (Vakulskas et al. 2015; Schoch et al. 2020).

*G. sulfurreducens* is an anaerobic electroactive bacterium, nonpathogenic, recognized as a prominent member of microbial communities involved in dissimilatory metal reduction and plays a significant role in biogeochemical cycles (Lovley 2012; Waite et al. 2020). This microorganism can oxidize simple organic compounds, such as acetate, as electron donors through the activity of enzymes including acetyl-CoA synthetase, converting these substrates to carbon dioxide. *G. sulfurreducens* utilizes a wide range of electron acceptors, including Fe(III), Mn(IV), U(IV), Co(III), Tc(VII), nitrate, and fumarate (Coppi et al. 2001; Methé et al. 2003). Through these metabolic processes, the bacterium facilitates the reduction and precipitation of soluble metal species (Lovley 2012; Lovley and Walker 2019). A defining characteristic of *G. sulfurreducens* is its capacity for extracellular electron transfer (EET), which supports redox transformations of metals in natural environments and offers substantial potential for environmental biotechnology, particularly for the remediation of heavy metal-contaminated sites and the development of bioelectrochemical systems (Ueki 2021; Lovley 2012). Two key components enabling EET in *G. sulfurreducens* are conductive pili, which function as extracellular nanowires, and *c*-type cytochromes, which mediate electron transfer (Walker et al. 2018).

In *G. sulfurreducens*, CsrA has been shown to regulate both biofilm formation and EET (Hernández-Eligio et al. 2025). However, no sRNAs from the CsrB/CsrC family that could regulate CsrA function have been characterized in *G. sulfurreducens*. Deletion of the *csrA* gene does not affect growth, but results in biofilms that are up to twice as thick as those produced by the wild-type (WT) strain. In addition, transcriptomic analysis using RNA sequencing (RNA-seq) revealed that approximately 357 genes exhibited altered RNA abundance in biofilms of the Δ*csrA* mutant grown on inert supports and electrodes in microbial fuel cells (MFCs). Among these genes, approximately 20% contain at least one putative CsrA binding site within the 5′ untranslated region of the mRNA. Furthermore, the Δ*csrA* mutant produces up to 40% more energy than the WT strain in MFCs (Hernández-Eligio et al. 2025).

Previous studies in *B. subtilis*, *Campylobacter jejuni*, and *C. difficile* have demonstrated that specific structural features of CsrA, including the C-terminal extension and particular amino acid residues, are essential for its interaction with FliW. In these bacteria, a conserved asparagine residue at position 55 (N55) in CsrA is required for the formation of the CsrA-FliW complex. Removal of the C-terminal extension or mutation of N55 to aspartate (N55D) abolishes the interaction between CsrA and FliW (Mercante et al. 2006; Mukherjee et al. 2016; Bogacz et al. 2021; Oshiro et al. 2021; Altagoer et al. 2016; Zhu et al. 2025). Similar to *B. subtilis*, *C. jejuni*, and *C. difficile*, CsrA from *G. sulfurreducens* possesses an extended C-terminal region and contains the conserved N55 residue. The CsrA protein from *G. sulfurreducens* shares 50%, 53%, and 46% sequence identity with homologs from *B. subtilis*, *Geobacillus thermodenitrificans*, and *E. coli*, respectively. These structural similarities, together with the established role of CsrA in regulating biofilm formation and extracellular electron transfer, underscore the importance of investigating its potential regulation by FliW in *G. sulfurreducens*.

In this study, we examined the electrochemical properties of biofilms produced by Δ*csrA*, Δ*fliW*, and WT strains of *G. sulfurreducens*. We also investigated whether CsrA interacts with FliW and, if so, whether the asparagine N55 is involved in this interaction. To address these questions, we proposed a regulatory framework in which the C-terminal region of CsrA interacts with FliW, thereby contributing to the control of electroconductive biofilm development and extracellular electron transfer in *G. sulfurreducens*.

## Materials and methods

### Growth conditions

The bacterial strains, plasmids, and oligonucleotides used in this study are summarized in Supplementary Table 1. *G. sulfurreducens* strains were grown anaerobically at 30 °C in NBAF medium with acetate (electron donor, 20 mM) and fumarate (electron acceptor, 40 mM) and acetate-Fe(III) citrate (20, and 50 mM, electron donor and acceptor, respectively) (Coppi et al. 2001). *E. coli* strains DH5α, BL21, SU101, SU202, and S17-1 were grown in LB medium at 37 °C. The *E. coli* strains were cultured in the presence of 200 µg/mL ampicillin (Ap^R^), 25 µg/ml of kanamycin (Km^R^), or 10 µg/mL tetracycline (Tc^R^), as appropriate. *G. sulfurreducens* strains were grown with 200 µg/mL Km^R^ and 50 µg/mL spectinomycin (Sp^R^). The *G. sulfurreducens* strains are stored in the strain bank of the Environmental Microbiology and Bioremediation Laboratory at the Institute of Biotechnology’s Department of Molecular Microbiology, under the direction of Dr. Katy Juárez.

### Plasmid construction

#### Plasmid for the construction of Δ*fliW* mutant strain

The pJCC plasmid was constructed to generate the Δ*fliW* mutant strain. The flanking regions of the *fliW* gene (1198 bp upstream and 1291 bp downstream) were amplified using the oligonucleotide pairs 1Fw/2Rev and 3Fw/4Rev (Supplementary Table 1), respectively, with genomic DNA as the template and Phusion High-Fidelity DNA Polymerase (Thermo Fisher Scientific, Vilnius, Lithuania). The two flanking regions were joined in a second round of PCR using the 1Fw/4Rev primer pair and Phusion DNA Polymerase. The resulting PCR product was digested with *Bam*HI and *Eco*RI and ligated into the corresponding sites of the pK18mobsacB cloning vector to generate pJCC. Restriction enzymes for DNA cloning were purchased from Thermo Fisher Scientific (Vilnius, Lithuania).

#### Plasmids for homodimerization and heterodimerization assay

The plasmids pSR658-csrA, pSR659-fliW, pSR658-fliW, and pSR659-csrA were constructed to test interactions between CsrA and FliW. The fragments corresponding to *csrA* (from the second codon to the stop codon) and *fliW* (in its entirety) were amplified by PCR using the oligonucleotide pairs FwcsrA/RvcsrA and FwfliW/RvfliW, respectively (Supplementary Table 1). The resulting PCR products (240 bp for *csrA* and 480 bp for *fliW*) were digested with *Xho*I and *Kpn*I and cloned into the pSR658 and pSR659 vectors digested with the same restriction enzymes. *Xho*I and *Kpn*I enzymes were also purchased from Thermo Fisher Scientific (Vilnius, Lithuania). All constructs were sequenced to confirm the correct open reading frame.

To identify the amino acid involved in the interaction between CsrA and FliW, the pSR658-csrA_N55D_ and pSR659-csrA_N55D_ plasmids were constructed using a site-directed mutagenesis protocol (Supplementary Table 1) (Zheng et al. 2004). Briefly, the *csrA*_N55D_ gene was amplified by PCR using Phusion high-fidelity Taq polymerase (Thermo Fisher Scientific, Vilnius, Lithuania) with the mutagenic oligonucleotides csrAN55DFw/csrAN55DRv and the plasmids pSR658-csrA and pSR659-csrA as templates, respectively (Supplementary Table 1; the bold letter indicates the codon substitution). The PCR products were digested with *Dpn*I to remove parental plasmid DNA and transformed into *E. coli* DH5α. The resulting plasmids were designated pSR658-csrA_N55D_ and pSR659-csrA_N55A_, and the mutations were confirmed by sequencing (data not shown).

#### Plasmid for CsrA protein expression and purification

The pET24a-csrA plasmid was constructed to express and purify the CsrA protein. A 254-bp fragment corresponding to the *csrA* gene was amplified from chromosomal DNA by PCR using Phusion high-fidelity DNA polymerase (Thermo Fisher Scientific, Vilnius, Lithuania) and the pETCsrAfw/pETCsrARv oligonucleotides (Supplementary Table 1). The PCR product was digested with *Nde*I and *Xho*I and ligated into the pET24a vector (Novagen). *E. coli* DH5α competent cells were transformed with the ligation mixture, and a transformant carrying the pET24a-csrA plasmid was isolated. The plasmid was sequenced to confirm the presence of an intact *csrA* gene fused to a polyhistidine-coding sequence.

### Complementation of Δ*fliW* mutant

The *fliW* gene was amplified using the NdeIfliWFw and HindfliWRv oligonucleotides (Supplementary Table 1, the cutting sites are underlined), chromosomal DNA, and Phusion Polymerase (Thermo Scientific, Vilnius, Lithuania). The amplified fragment (450-bp), flanked with *Nde*I/*Hind*III restriction sites, was cloned into the same sites of the pJET-RRflg plasmid (Hernández-Eligio et al. 2025). The obtained plasmid was named pJET-RRflg-*fliW*. This plasmid was digested with *Bam*HI and *Hind*III to release the RRflg-*fliW* gene, and this fragment was cloned into the same sites of the pRG5.1 plasmid (Kim et al. 2005). The resulting plasmid containing the *fliW* gene was sequenced and named pRG5.1-RRflg-fliW. The plasmid was subsequently electroporated into the Δ*fliW* mutant strain, and the resulting Sp^R^-resistant colonies were isolated to confirm the presence of the plasmid by extracting the plasmid DNA followed by digestion with *Bam*HI/*Hind*III restriction enzymes.

### Construction of Δ*fliW* mutant in *G. sulfurreducens*

The Δ*fliW* mutant strain was constructed using a markerless deletion protocol (Chan et al. 2015). This method removes the *fliW* coding region without altering upstream or downstream chromosomal regions. The pJCC plasmid was transformed into the *E. coli* conjugative donor strain S17-1 for transfer into the *G. sulfurreducens* recipient. Briefly, 1 mL of a fully grown *G. sulfurreducens* acetate-fumarate culture was pelleted onto 1 mL of an S17-1 culture carrying pJCC. The mixture was placed on 0.22-µm-pore-size filters resting on acetate-fumarate agar plates under anaerobic conditions and incubated for 4 h. After incubation, cells were streaked onto acetate-fumarate anaerobic plates containing Km^R^. This procedure allowed selection of *G. sulfurreducens* strains with pJCC integrated into either flanking region of the *fliW* gene. A scarless deletion mutant was selected on acetate-fumarate anaerobic plates supplemented with 10% sucrose and confirmed by PCR using primers flanking the deletion site (Supplementary Fig. 1).

### Confocal laser scanning microscopy

Biofilm structure and the ratio of live to dead cells were analyzed using confocal laser scanning microscopy (CLSM). Fluorine-doped tin oxide (FTO) electrodes were used as substrates for biofilm formation inside hermetically sealed test tubes under anaerobic conditions with NBAF medium. Incubation was carried out without shaking at 25 °C for 48 h and 72 h. All solutions used were sterile and anaerobic. After removal from the culture medium, planktonic cells were gently removed from the biofilms using a solution containing 0.002 M cysteine and 0.9% isotonic saline. Biofilms were then stained with the LIVE/DEAD® BacLight Bacterial Viability Kit (Invitrogen, Thermo Fisher Scientific, Eugene, Oregon, USA) using a mixture of 0.00334 mM SYTO9 and 0.02 M propidium iodide dissolved in 0.9% saline and 0.1 M cysteine. Samples were incubated with the dye for 10 min in the dark, washed with 0.002 M cysteine and 0.9% saline, and imaged using an Olympus FV1000 microscope. Excitation wavelengths of 488 nm (green channel) and 559 nm (red channel) were used with an immersion objective (LUMFLN 60 ×/1.1 W). Fluorescence was detected at 500–545 nm for the green channel and 570–670 nm for the red channel. Images were acquired along the Z-axis at regular intervals. Image analysis was performed using Comstat2 (version 2.1) and Fiji (version 2.9.0) (Heydorn et al. 2000; Schindelin et al. 2012).

### Expression and purification of CsrA protein

Expression of CsrA-6His in *E. coli* BL21/pET24a-csrA was induced at the mid-exponential phase (A600 nm 0.5–0.6) by addition of IPTG (1 mM). After 4 h of induction, protein purification was performed at 4 °C under non-denaturing conditions using a HisPur Ni-NTA resin column according to the manufacturer’s instructions (Thermo Fisher Scientific, Vilnius, Lithuania). The purified protein was maintained in storage buffer (10 mM Tris-HCl, 50 mM potassium chloride, pH 8.0), concentrated using Microcon YM-10 centrifugal filters (Amicon), and stored at 4 °C. Protein concentrations were determined using the Bradford assay (Bio-Rad, California, USA) with BSA as a standard. The integrity of the purified fusion protein (approximately 9.9 kDa) was confirmed by SDS-PAGE and western blot analysis.

### CsrA homodimerization assay

To test CsrA homodimerization, *E. coli* SU101 reporter strain cells were transformed with the pSR658 or pSR658-csrA plasmids, which carries a chromosomal *sulA–lacZ* transcriptional fusion (Dimitrova et al. 1998; Daines and Silver 2000). The plasmid pSR658-HilD1, expressing the LexA_DBDwt_-HilD fusion protein, was used as an interaction control since HilD homodimerization has been previously confirmed (Paredes-Amaya et al. 2018). One percent of the transformants culture cells were grown overnight and were inoculated into LB medium supplemented with Tc^R^ and 1 mM IPTG to induce the expression of LexA_DBDwt_-CsrA and LexA_DBDwt_-HilD fusion proteins. Cultures were harvested at an A600 of 1.0 and used for β-galactosidase activity assays.

### CsrA-FliW heterodimerization assay

In vivo interactions between CsrA and FliW were examined using the *E. coli* SU202 reporter strain, which carries a *sulA–lacZ* transcriptional fusion with a hybrid LexA operator (Dimitrova et al. 1998; Daines and Silver 2000). SU202 cells were first transformed with pSR658 or pSR658-csrA and subsequently transformed with pSR659 or pSR659-fliW. The combination of pSR658-fliW with pSR659-csrA was also tested. The plasmids pSR658-HilD1 and pSR659-HilE1, which express the fusion proteins LexA_DBDwt_-HilD and LexA_DBDmut_-HilE, respectively, and whose interaction has been previously reported, were used as interaction controls (Paredes-Amaya et al. 2018). Transformants were grown in LB with Tet^R^ and Ap^R^ and with 1 mM IPTG to induce expression of LexA_DBDwt_-CsrA and LexA_DBDmut_-FliW fusion proteins or LexA_DBDwt_-FliW and LexA_DBDmut_-CsrA. Cultures were collected at an A600 of 1.0 and assayed for β-galactosidase activity.

### Pull-down assays

Pull-down assays were performed as previously described (Paredes-Amaya et al. 2018) using purified CsrA-6His protein and whole-cell extracts from *E. coli* SU101 expressing LexA_DBDwt_ and LexA_DBDwt_-FliW. To immobilize the bait protein, 15 µg of purified CsrA-6His was incubated for 1 h at 10 °C with 100 µL of HisPur resin (Thermo Fisher Scientific, Vilnius, Lithuania), previously equilibrated with equilibration buffer (20 mM sodium phosphate, 300 mM sodium chloride, and 10 mM imidazole) in a 2-mL microcentrifuge tube. The resin containing immobilized CsrA-6His was washed with 1 mL of wash buffer (20 mM sodium phosphate, 300 mM sodium chloride, and 25 mM imidazole) by centrifugation at 4000 × g for 1 min. After careful removal of the supernatant, the resin was incubated for 1 h at 10 °C with 100 µL of whole-cell extract from SU101, SU101/LexA_DBDwt_, or SU101/LexA_DBDwt_-FliW. The resin was washed five times with 1 mL of wash buffer to remove unbound proteins by centrifugation at 4000 × g for 1 min. Bound bait and prey proteins were eluted with 100 µL of elution buffer (20 mM sodium phosphate, 300 mM sodium chloride, and 250 mM imidazole), mixed with SDS-PAGE loading buffer, and boiled for 10 min. The samples were subsequently analyzed by western blotting.

### β-Galactosidase assay

β-Galactosidase activity from liquid *E. coli* cultures was assayed as previously described (Price-Carter et al. 2001).

### Western blot assays

Western blot assays were performed for the immunodetection of LexA fusion proteins, 6 × histidine-tagged proteins, and maltose-binding protein (MBP). We used MBP-detection as a loading control in Western blot assays. Whole-cell extracts were prepared from *E. coli* samples collected after 4 h of induction with 1 mM IPTG. Proteins were separated by 15% SDS–PAGE and transferred onto nitrocellulose membranes (Merck-Millipore) using a TE22 Mighty Small Transfer Tank (Hoefer, Bridgewater, USA). Transfer efficiency was monitored by Ponceau red staining (Sigma-Aldrich, USA). The membranes were blocked overnight at 4 °C with 5% low-fat dry milk and then thoroughly washed with PBS-Tween (0.3%) followed by PBS. The membranes were then incubated overnight at 4 °C with gentle agitation with the following primary antibodies diluted in PBS-BSA (0.3%): anti-LexA (1:1000; Sigma-Aldrich, USA), anti-6 × histidine (1:5000; Sigma-Aldrich, China), and anti-MBP (1:5000; Santa Cruz Biotechnology, Texas, USA). After washing with PBS-Tween (0.3%) and PBS, the membranes were incubated overnight at 4 °C with alkaline phosphatase-conjugated anti-rabbit secondary antibodies (Sigma-Aldrich, AP132A) diluted 1:10,000 in PBS-BSA (0.3%). Finally, the membranes were developed using 1 mL of BCIP/NBT solution (Sigma-Aldrich, USA).

### RT-qPCR

Total RNA was extracted from *G. sulfurreducens* DL1 and Δ*fliW* strains and used for RT-qPCR analyses. RNA was isolated from *G. sulfurreducens* cells recovered from biofilms after 48 h of incubation on glass using the RNeasy Mini Kit (Qiagen, Hilden, Germany), following the manufacturer’s instructions. Genomic DNA contamination was removed by treatment with DNase I (Thermo Fisher Scientific, Vilnius, Lithuania). RNA concentration and purity were measured using a NanoDrop 2000c spectrophotometer (Thermo Scientific, USA), and RNA integrity was verified by 0.8% agarose gel electrophoresis. cDNA was synthesized using the RevertAid First Strand DNA Synthesis Kit (Thermo Fisher Scientific, Vilnius, Lithuania) and gene-specific reverse oligonucleotides (Table S1). RT-qPCR was performed using the Maxima SYBR Green/ROX qPCR Master Mix (Thermo Fisher Scientific, Vilnius, Lithuania) on a Rotor-Gene Q 2plex HRM instrument (Qiagen, Hilden, Germany). Relative expression levels of target genes were calculated using the 2^−ΔΔCT^ method with Rotor-Gene Q Series Software. Expression of the *gsu2822* gene was used as the internal control. The experiments were conducted twice, each with three replicates, and mean values were calculated.

### Electrochemical methods

All electrochemical studies were conducted in a conventional three-electrode cell with an Ag/AgCl reference electrode (*E* = 0.199 V vs. the standard hydrogen electrode [SHE]); all potentials reported herein are referenced to the SHE. A platinum mesh served as the counter electrode, and biofilms grown on FTO electrodes were used as the working electrodes. After 48 and 72 h of incubation, the biofilm-coated electrodes were carefully removed from the sealed test tubes and placed in the electrochemical cell. The cell consisted of a hermetically sealed glass chamber continuously bubbled with an N_2_:CO_2_ (80:20) gas mixture. Basal medium (BM) (Hernández-Eligio et al. 2020) was used as the electrolyte. Open-circuit potential (OCP), cyclic voltammetry (CV), and square-wave voltammetry (SWV) were used for electrochemical characterization (Richter et al. 2009). OCP was recorded for 10 min. CV measurements were performed under both non-catalytic and catalytic conditions with the addition of 20 mM sodium acetate (NaAc) over a potential window from − 0.6 to 0.5 V at a scan rate of 0.002 V s^−1^. SWV was carried out with a step potential of 0.001 V, a modulation amplitude of 0.01 V, and a frequency of 37 Hz. Scans were performed from negative to positive potentials over the same potential window used for CV. All electrochemical studies were performed in an Metrohm Dropsens potentiostat/galvanostat (Metrohm, Asturias, Spain), and data were acquired using the Nova 2.1 software.

To further analyze the kinetic behavior of the biofilms, electron transfer reactions were described using a simplified model based on the Butler-Volmer equation (Vetter 2013). Voltammetric curves were interpreted using the Nernst-Monod equation to account for the effects of substrate concentration and anode potential (Marcus et al. 2007; Torres et al. 2008, 2010). Experimental data were fitted to predicted values by minimizing the residual sum of squares (RSS) using nonlinear least-squares estimation. Minimization was performed with the scaled Levenberg–Marquardt algorithm (tolerance = 0.0001) implemented in QtiPlot 0.9.8.9 – svn 2288 (Copyright 2004–2020 IonVasiliev).

### Statistical analysis

Data from β-galactosidase assays were analyzed using one-way analysis of variance (ANOVA) followed by Tukey’s multiple comparison test. A *p*-value of < 0.05 was considered statistically significant. All analyses were performed using Prism 10 (version 10.5.0; GraphPad Software, LLC).

## Results

### CsrA dimerization in *G. sulfurreducens*

**Fig. 1:**
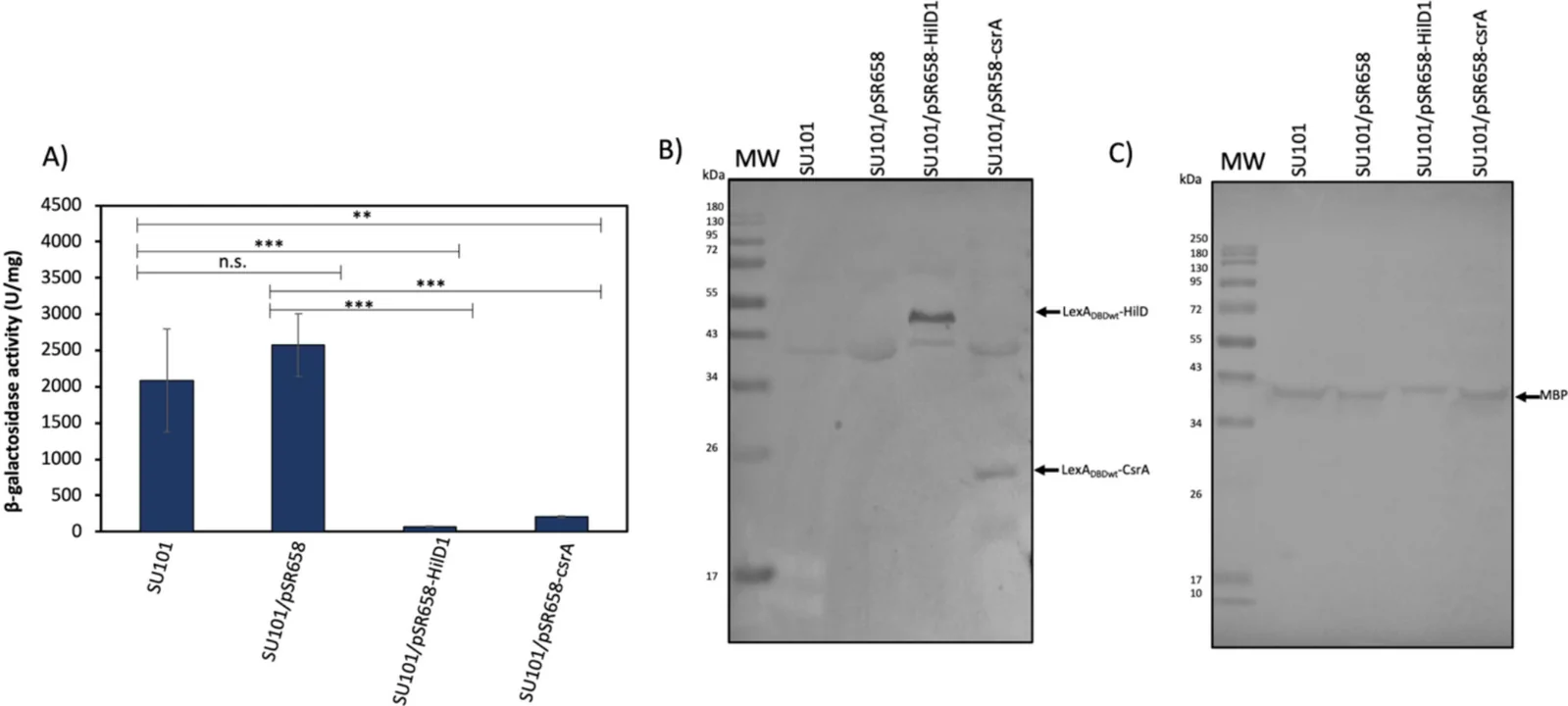
CsrA of *G. sulfurreducens* form homodimers. **A** β-Galactosidase activity in the *E. coli* SU101 reporter strain containing pSR658 (LexA_DBDwt_), pSR658-HilD (LexA_DBDwt_-HilD), and pSR658-csrA (LexA_DBDwt_-CsrA) plasmids. Data represent the averages of three independent experiments performed in triplicate, with bars indicating standard deviations. ***, statistically significant difference compared to the absence of CsrA (*p* < 0.001); **, statistically significant difference compared to the absence of CsrA (*p* < 0.01); n.s., not significant. **B** Western blot analysis of LexA_DBDwt_, LexA_DBDwt_-HilD, and LexA_DBDwt_-CsrA using anti-LexA antibodies. Arrows indicate the expected bands for LexA_DBDwt_-HilD (45 kDa) and LexA_DBDwt_-CsrA (18.2 kDa). MW, protein molecular weight standards (color prestained protein standard, broad range 10–250 kDa, NEB). **C** Loading control showing expression of MBP detected using anti-MBP antibodies

To determine whether *G. sulfurreducens* CsrA forms dimers, we used a LexA-based genetic system for homodimerization in *E. coli* SU101 (Dimitrova et al. 1998; Daines and Silver 2000). The pSR658-csrA plasmid expressing LexA_DBDwt_-CsrA was compared with positive (LexA_DBDwt_-HilD) and negative controls (empty vector and SU101 without plasmid). β-Galactosidase activity was decreased in SU101/pSR658-csrA and SU101/pSR658-HilD strains compared to controls (Fig. 1A). Western blot analysis confirmed expression of the fusion proteins LexA_DBDwt_-HilD (~ 45 kDa) and LexA_DBDwt_-CsrA (~ 18.2 kDa) (Fig. 1B).

### FliW interacts with CsrA

**Fig. 2:**
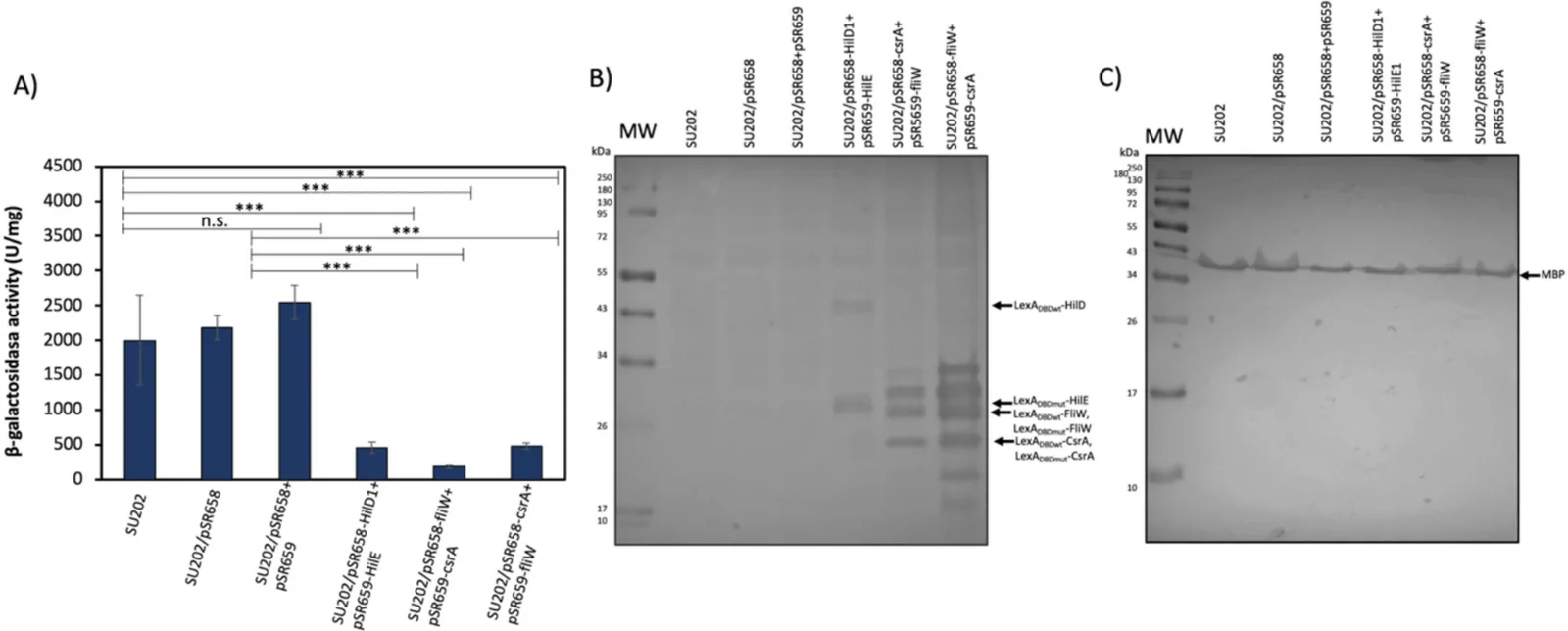
CsrA and FliW of *G. sulfurreducens* interact in the LexA-based genetic system. **A** β-Galactosidase activity in the *E. coli* SU202 reporter strain containing the pSR658 (LexA_DBDwt_); pSR659 (LexA_DBDmut_); pSR658-HilD (LexA_DBDwt_-HilD) and pSR659-HilE1 (LexA_DBDmut_-HilE); pSR658-csrA (LexA_DBDwt_-CsrA) and pSR659-fliW (LexA_DBDmut_-FliW); pSR658-fliW (LexA_DBDwt_-FliW) and pSR659-csrA (LexA_DBDmut_-CsrA) plasmids. Data are averages of three independent experiments performed in triplicate; bars indicate standard deviations. ***, statistically significant difference compared with the absence of CsrA and FliW or HilD and HilE proteins (*p* < 0.001); n.s., not significant. **B** Western blot analysis of LexA_DBDwt_, LexA_DBDwt_-HilD, LexA_DBDmut_-HilE, LexA_DBDwt_-CsrA, LexA_DBDmut_-FliW, LexA_DBDwt_-FliW, and LexA_DBDmut_-CsrA using anti-LexA antibodies. The arrows indicate the expected bands for LexA_DBDwt_-HilD (45 kDa), LexA_DBDmut_-HilE (30 kDa), LexA_DBDwt_-CsrA and LexA_DBDmut_-CsrA (18.2 kDa), and LexA_DBDmut_-FliW and LexA_DBDwt_-FliW (27 kDa). MW, protein molecular weight standards (color prestained protein standard, broad range 10–250 kDa, NEB). **C** Loading control showing expression of MBP detected using anti-MBP antibodies

**Fig. 3:**
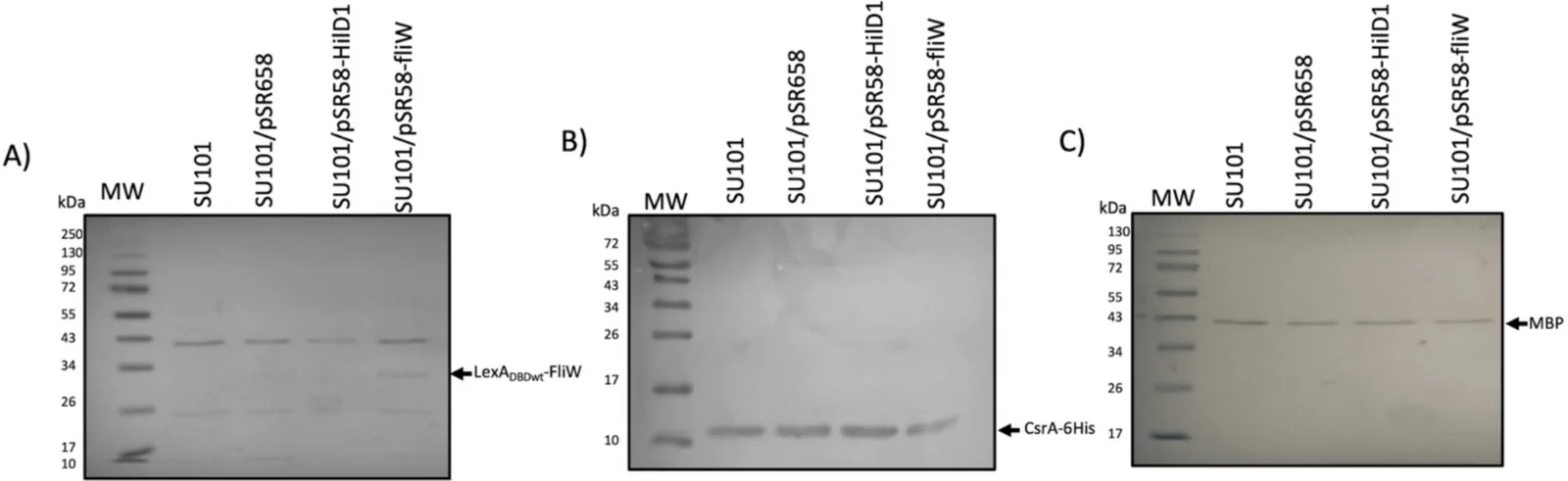
CsrA interacts with FliW in pull-down assays. **A** Western blot analysis of pull-down assays using CsrA-6His as bait and LexA_DBD_ fusion proteins as prey, detected with anti-LexA antibodies. Arrows indicate the expected band for LexA_DBDmut_-FliW (27 kDa). **B** Western blot analysis detecting CsrA-6His using anti-6His antibodies. **C** Loading control showing MBP expression detected using anti-MBP antibodies. MW, protein molecular weight standards (color prestained protein standard, broad range 10–250 kDa, NEB)

Heterodimerization assays using the LexA-based system in *E. coli* SU202 showed a decrease in *sulA*–*lacZ* expression in strains expressing LexA_DBDwt_-CsrA/LexA_DBDmut_-FliW and LexA_DBDwt_-FliW/LexA_DBDmut_-CsrA, similar to positive controls (Fig. 2A). Western blot analysis confirmed the presence of all tested fusion proteins (Fig. 2B). To confirm the interaction between CsrA and FliW, a pull-down assay was performed using CsrA fused to a 6-histidine tag (CsrA-6His) as bait. CsrA-6His was first immobilized on HisPur Ni-NTA resin. Soluble whole-cell extracts from the SU101 strain—either without plasmid, carrying the empty plasmid pSR658, expressing LexA_DBDwt_, expressing LexA_DBDwt_-HilD (pSR658-HilD1), or expressing LexA_DBDwt_-FliW (pSR658-fliW)—were then incubated with the resin. Following multiple washes, bound proteins were eluted and analyzed by western blot using anti-LexA_DBD_ or anti-6His antibodies. Pull-down assays using CsrA-6His as bait further confirmed the direct interaction between CsrA and FliW (Fig. 3).

### Role of Asn55 in CsrA-FliW interaction

**Fig. 4:**
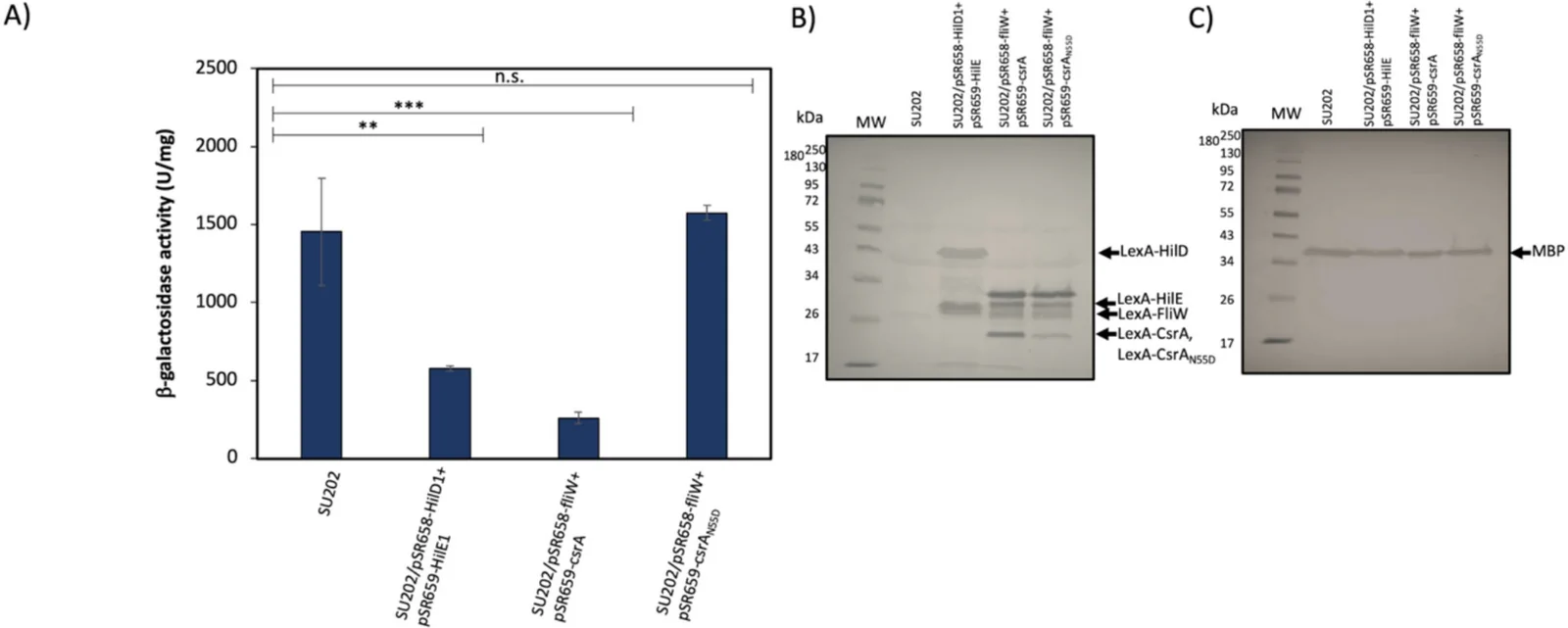
The asparagine 55 in CsrA is critical for CsrA/FliW heterodimer formation. **A** β-Galactosidase activity in the *E. coli* SU202 reporter strain containing the pSR658-HilD (LexA_DBDwt_-HilD) and pSR659-HilE1 (LexA_DBDmut_-HilE); pSR658-fliW (LexA_DBDwt_-FliW) and pSR659-csrA (LexA_DBDmut_-CsrA); and pSR658-fliW (LexA_DBDwt_-FliW) and pSR659-csrA_N55D_ (LexA_DBDmut_-CsrA_N55D_) plasmids. The data represent averages of three independent experiments performed in triplicate; bars indicate standard deviations. ***, statistically significant difference compared to the absence of CsrA and FliW (*p* < 0.001); **, statistically significant difference compared to the absence of HilD and HilE (*p* < 0.01); n.s., no significant difference. **B** Western blot analysis of LexA_DBDwt_-HilD, LexA_DBDmut_-HilE, LexA_DBDwt_-FliW, LexA_DBDmut_-CsrA, and LexA_DBDmut_-CsrA_N55D_ using anti-LexA antibodies. The arrows indicate expected bands for LexA_DBDwt_-HilD (45 kDa), LexA_DBDmut_-HilE (30 kDa), LexA_DBDmut_-CsrA and LexA_DBDmut_-CsrA_N55D_ (18.2 kDa), and LexA_DBDwt_-FliW (27 kDa). MW, protein molecular weight standards (color prestained protein standard, broad range 10–250 kDa, NEB). **C** Loading control showing MBP expression detected using anti-MBP antibodies

To assess whether this residue is required for the interaction between CsrA and FliW in *G. sulfurreducens*, the asparagine residue at position 55 was substituted with aspartic acid to generate the CsrA_N55D_ variant, and the interaction of this mutant protein with FliW was evaluated using the LexA-based genetic system described in the Materials and Methods. The pSR659-csrA_N55D_ plasmid, expressing LexA_DBDmut_-CsrA_N55D_, was constructed and tested in combination with pSR658-fliW, expressing LexA_DBDwt_-FliW. Heterodimerization assays were then performed to examine the interaction between CsrA_N55D_ and FliW. Plasmids pSR658-HilD1 and pSR659-HilE1, expressing LexA_DBDwt_-HilD and LexA_DBDmut_-HilE, and pSR658-fliW and pSR659-csrA, expressing LexA_DBDwt_-FliW and LexA_DBDmut_-CsrA, were used as a positive heterodimerization controls. The SU102 strain without any plasmid was used as negative control. As expected, *sulA*–*lacZ* was expressed in the SU202 control strain, whereas repression of *sulA–lacZ* expression occurred in the SU202/pSR658-HilD1 + pSR659-HilE1 and SU202/pSR658-fliW + pSR659-csrA strains (Fig. 4A). In contrast, *sulA*–*lacZ* expression was restored in the SU202/pSR658-fliW + pSR659-csrA_N55D_ strain, indicating a loss of interaction between FliW and the CsrA_N55D_ variant. Western blot confirmed the presence of both fusion proteins (Fig. 4B).

### Impact of the FliW mutant on growth using fumarate and Fe(III) as electron acceptors and on the expression of selected genes

**Fig. 5:**
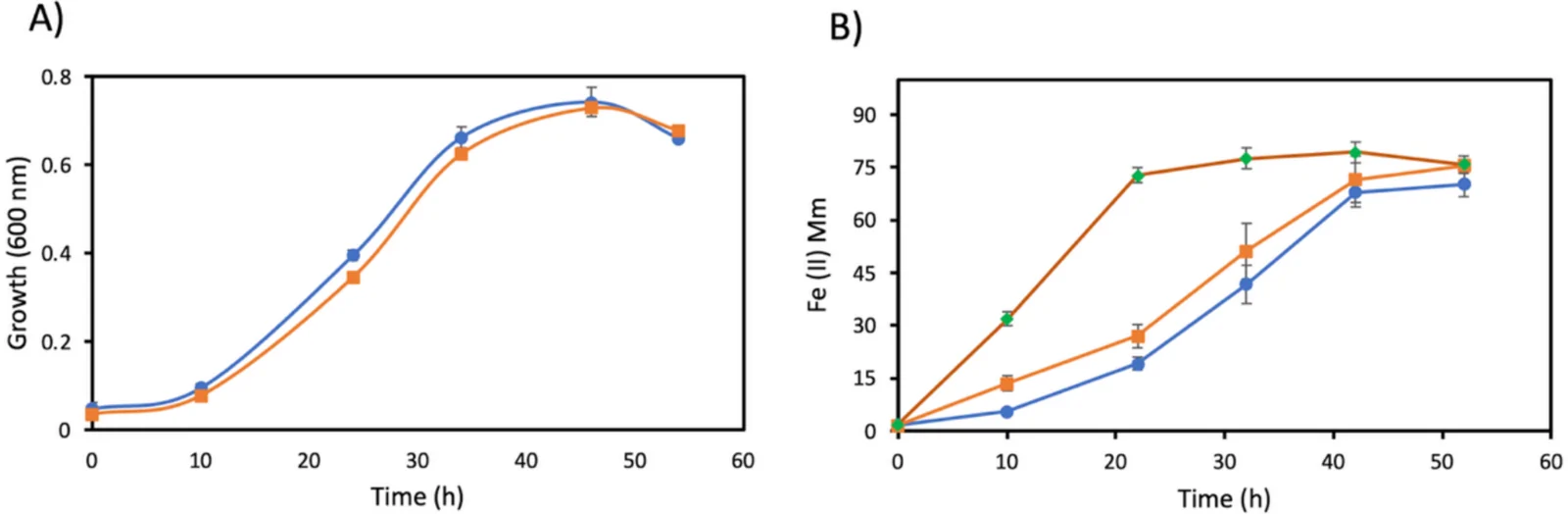
Growth and Fe(III) reduction in *G. sulfurreducens* Δ*fliW* strain. **A** Growth curves of WT (blue line with circles) and Δ*fliW* mutant strain (orange line with squares). **B** Soluble Fe(III) reduction by WT (blue line with circles), Δ*fliW* mutant (orange line with squares), and Δ*csrA* mutant (red line with squares)

The deletion of *fliW* did not affect growth using -fumarate or soluble Fe(III) as electron acceptors, compared to the growth of the WT strain (Fig. 5A-B). Furthermore, to investigate whether FliW deficiency impacts the transcript levels of genes previously associated with CsrA activity, we quantified the expression of *gsu3268*, *gsu3274*, *gsu2236*, *gsu0972*, *gsu1944*, *gsu3014*, *gsu0597*, and *gsu0018* via RT-qPCR in biofilms of the Δ*fliW* and WT strains. Transcription levels of *gsu3268*, *gsu3274*, and *gsu0972* were reduced in the Δ*fliW* strain, while these genes were upregulated in the Δ*csrA* strain. In contrast, *gsu1944*, *gsu3014*, and *gsu0597* exhibited a higher abundance in the Δ*fliW* strain and reduced levels in the Δ*csrA* strain, suggesting that FliW modulates the CsrA-dependent transcriptomic profile. Additionally, *gsu2236* and *gsu0018* showed similar expression trends in Δ*fliW* and Δ*csrA* strains, potentially reflecting indirect effects or additional regulatory mechanisms independent of the FliW-CsrA interaction (Table 1).

**Table 1:** RT-qPCR analysis of selected genes expressed in *G. sulfurreducens* Δ*fliW* formed biofilm

Locus ID	Description	Functional category	Avg Δ*fliW*/Avg DL1	Regulation in Δ*csrA*
*gsu3268*	*feoB-2*, ferrous iron transport protein B	Transport	0.56 ± 0.05 *	Upregulation
*gsu3274*	Cytochrome, 1 heme-binding site	Energy metabolism	0.45 ± 1.03 *	Upregulation
*gsu2236*	*relA*, GTP di-phosphokinase	Nucleotide metabolism	1.58 ± 0.11 *	Upregulation
*gsu0972*	ATPase, AAA family	Transport	0.25 ± 0.22 *	Upregulation
*gsu1944*	PEP motif-containing protein	Proteolysis	13.03 ± 3.09 *	Downregulation
*gsu3014*	Metal-dependent phosphohydrolase	Others	8.04 ± 1.37 *	Downregulation
*gsu0597*	Hypothetical protein	Unknown function	1.16 ± 0.29 *	Downregulation
*gsu0018*	Transcriptional regulation, GntR family/aminotransferase class-I	Transcriptional and regulatory functions	0.56 ± 0.26 *	Downregulation

Asterisks indicate statistically significant differences in gene expression between Δ*fliW* and wild-type biofilms (*p* < 0.05, two-tailed Student’s *t*-test)

### Effect of FliW on biofilm formation

**Fig. 6:**
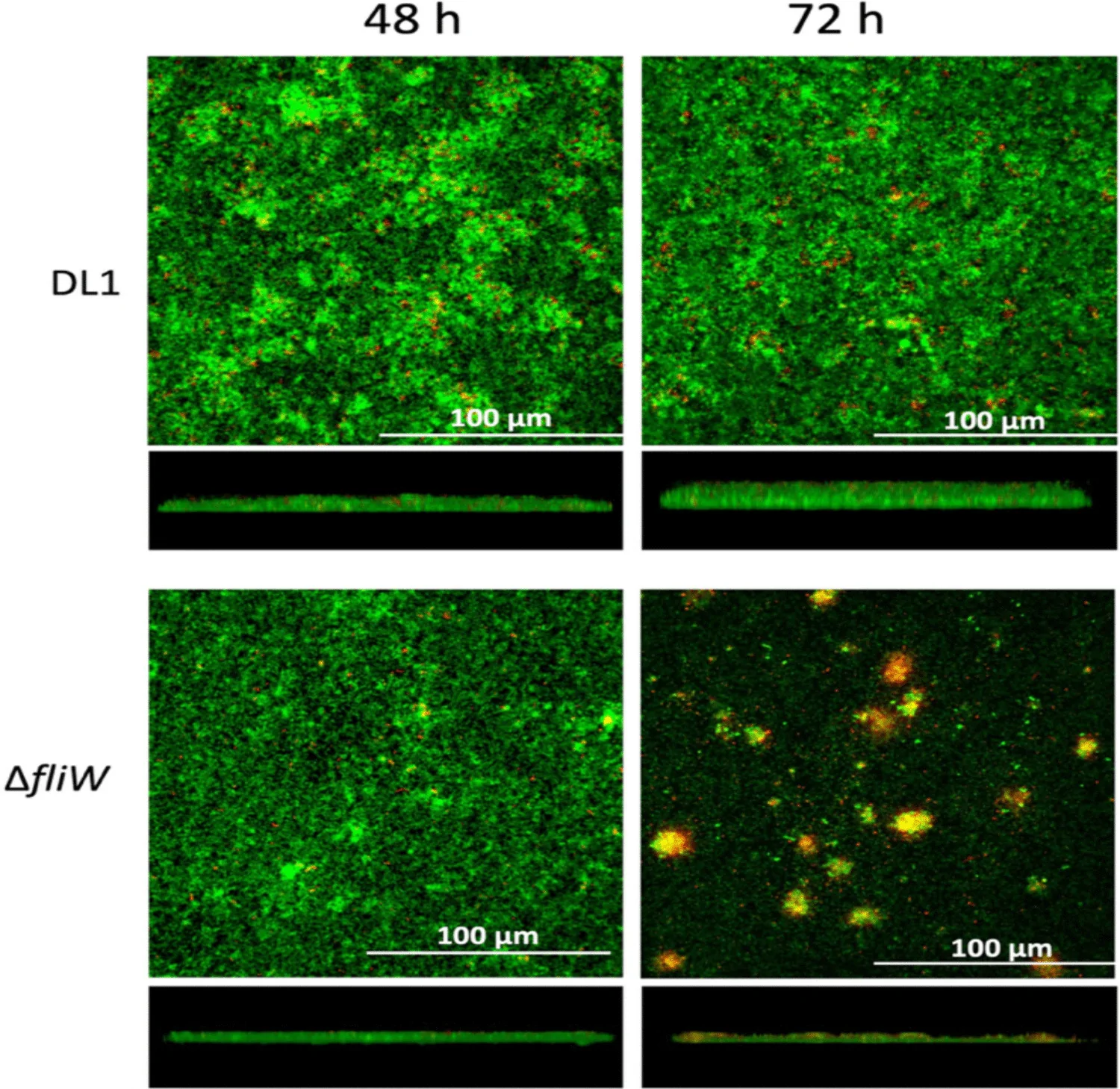
Characterization of Δ*fliW* and wild-type biofilms. CLSM images of Δ*fliW* and WT biofilms grown on FTO electrodes in acetate-fumarate medium. The top and bottom panels show top and side view projections of DL1 (wild type) and Δ*fliW* biofilms at 48 h and 72 h. Live cells are indicated in green, and dead cells are shown in red. Red spots correspond to dead cells

To assess the impact of *fliW* deletion on biofilm formation, we analyzed biofilms formed by the WT and Δ*fliW* strains grown on FTO electrodes in acetate-fumarate medium for 48 and 72 h using CLSM. Biofilms produced by the WT strain exhibited a modest increase in thickness over time, from 14.5 µm (± 2.12) at 48 h to 17 µm (± 4.24) at 72 h (Fig. 6A). Although the number of dead cells increased at 72 h, the proportion of viable cells remained high throughout the experiment, exceeding 75% at both time points (81.15 ± 3.75 at 48 h and 77.47 ± 4.37 at 72 h). In contrast, the Δ*fliW* strain formed thinner biofilms that did not increase in thickness over time, measuring 13 µm (± 4.24) at 48 h and 11.5 µm (± 3.53) at 72 h (Fig. 6B). Moreover, Δ*fliW* biofilms displayed a higher abundance of dead cells than those of the WT strain, a phenotype that became more pronounced at 72 h. Consistent with this observation, cell viability in the Δ*fliW* biofilms declined sharply over time, decreasing from 90.37 ± 2.16 at 48 h to 26.33 ± 4.34 at 72 h. CLSM analysis revealed that Δ*fliW* biofilms were thinner and displayed reduced cell viability at 72 h compared to WT (Fig. 6). Exopolysaccharides are major components of the biofilms formed by *G. sulfurreducens* (Rollefson et al. 2011). Therefore, the production of exopolysaccharides present in the biofilms was compared and quantified using crystal violet staining. As shown in the absorbance measurements in Supplementary Fig. 2, the *fliW* mutant strain exhibits a 50% reduction in exopolysaccharides compared to the wild-type strain at 72 h. While the Δ*fliW* strain exhibits a decrease in biofilm production, the Δ*csrA* mutant strain produces up to twice as much biofilm as the wild-type strain, as described above (Hernández-Eligio et al. 2025). To confirm that the negative phenotype in cell viability and exopolysaccharide production in the Δ*fliW* biofilms strain was due to inactivation of the *fliW* gene, we carried out the complementation of this mutant strain. The pRG5.1 or pRG5.1-RRflg-fliW plasmids were used to transform the Δ*fliW* strain. Cell viability in Δ*fliW* biofilms was complemented to wild-type levels in the presence of pRG5.1-RRflg-fliW plasmid, but not with the pRG5.1 (empty vector) (Supplementary Fig. 3). On the other hand, exopolysaccharide production in the Δ*fliW*/pRG5.1-RRflg-fliW strain was partially restored to wild-type levels, but not in the Δ*fliW*/pRG5.1 strain (Supplementary Fig. 2). This indicates that, in the absence of FliW, CsrA activity is associated with altered levels of transcripts involved in biofilm formation, potentially reflecting both direct and indirect regulatory effects.

### Electrochemical characterization of biofilms

OCP, CV, and SWV analyses were conducted to characterize the electrochemical properties of Δ*csrA*, WT (DL1), and Δ*fliW* biofilms. The Δc*srA* mutant was previously constructed and validated by Hernández-Eligio et al. (2025) using the protocol described by Chan et al. (2015). All biofilms were grown without an applied potential for 48 or 72 h. Electrochemical measurements were conducted in a non-nutrient buffer to assess intrinsic biofilm properties, minimizing medium effects. The average OCP values after 10 min of stabilization are shown in Supplementary Fig. 4A. Bare FTO electrodes exhibited an OCP of approximately 0.2 V, whereas WT biofilms displayed OCPs between − 0.15 and − 0.25 V. In contrast, Δ*csrA* biofilms exhibited much more negative potentials OCPs (≈ − 0.3 V), both with and without acetate. The Δ*fliW* mutant displayed OCPs similar to WT biofilms. Additionally, a more detailed description of these results is shown in Supplementary Fig. 4.

**Fig. 7:**
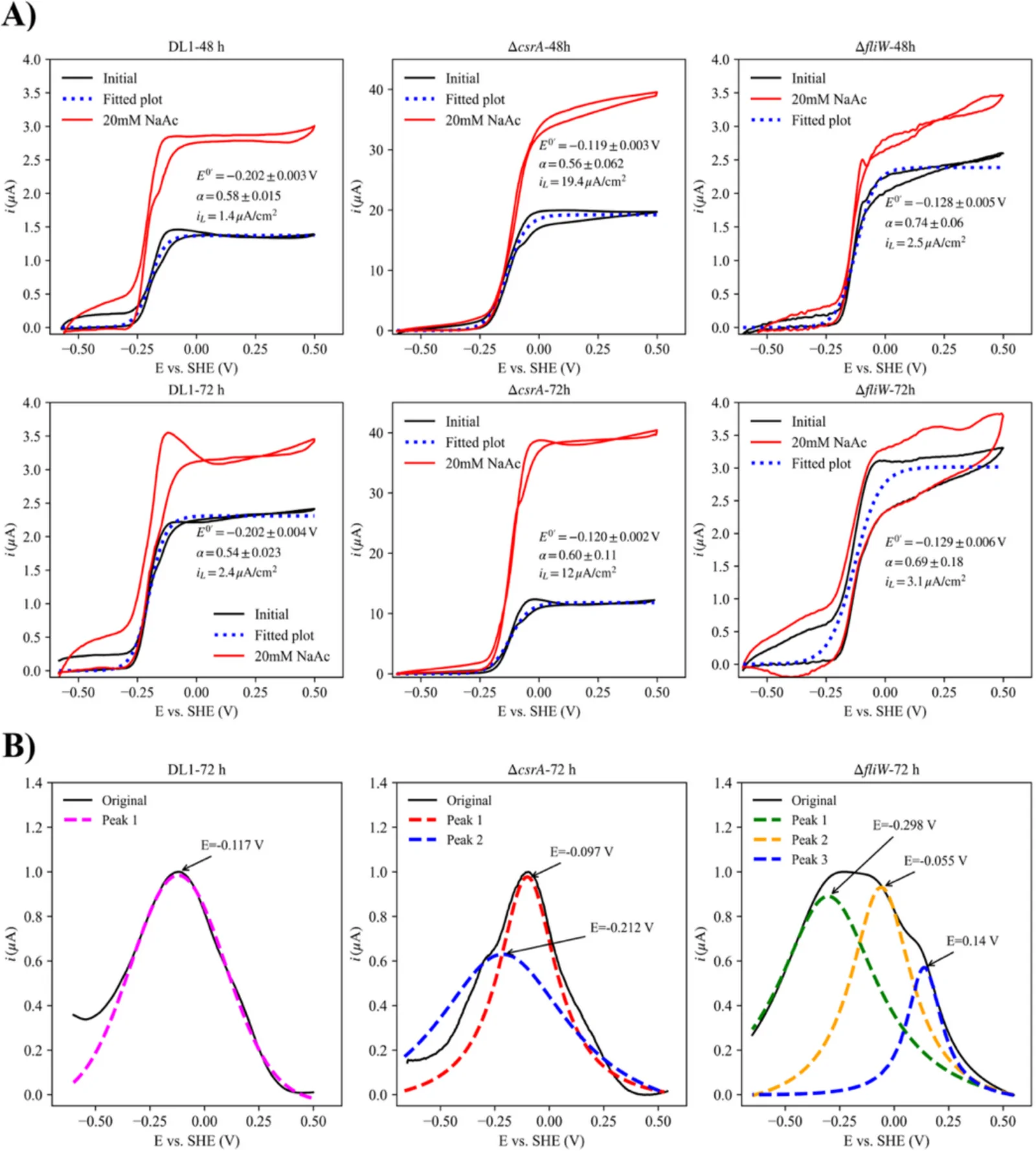
Electrochemical characterization of wild-type (DL1), Δ*csrA*, and Δ*fliW G. sulfurreducens* biofilms. **A** Cyclic voltammetry (CV) at a 1 mV/s scan rate for DL1, Δ*csrA*, and Δ*fliW* biofilms on FTO electrodes. Red lines indicate the electrochemical response after the addition of NaAc, and blue dotted lines represent the initial voltammogram fitted to the Nernst-Monod equation. **B** Square wave voltammetry (SWV) of DL1, Δ*csrA*, and Δ*fliW* biofilms on FTO electrodes at 72 h, using a 0.001 V/s scan rate and 0.01 V amplitude. Dotted lines correspond to multi-peak Lorentzian fits

Figure 7A shows the CV responses of the biofilms with and without NaAc. All three strains exhibited sigmoidal voltammograms on FTO electrodes, indicating electroactivity and extracellular electron transfer. Voltammograms were analyzed using the Nernst-Monod model (Supplementary Fig. 5), which relates electron-donor utilization (NaAc) to electrical potential. A notable 100 mV difference in formal potential (E^0^′) was observed between strains. First-derivative analysis confirmed this result (Supplementary Fig. 6). Limiting current values (*i*_*L*_) further confirmed the superior electroactivity of Δ*csrA* biofilms. Without acetate, Δ*csrA* currents were 13- and 5-fold higher than DL1 and Δ*fliW* after 48 h and 72 h, respectively. With acetate, Δ*csrA* biofilms reached ~ 40 µA, approximately 13 times greater than DL1 (~ 3 µA). Figure 7B illustrates the SWV responses in the presence of NaAc. WT (DL1) biofilms exhibited a redox peak at − 0.117 V, fitted using Lorentzian curves via the Levenberg-Marquardt algorithm in QtiPlot 0.9.8.9. Mutant biofilms showed distinct peaks: Δ*csrA* at − 0.097 V and − 0.212 V, and Δ*fliW* at − 0.298 V, − 0.55 V, and 0.14 V.

## Discussion

CsrA is a central post-transcriptional regulator that controls multiple cellular processes in bacteria, including biofilm formation and extracellular electron transfer. In *G*. *sulfurreducens*, deletion of *csrA* enhances biofilm formation, with the mutant exhibiting biofilm thicknesses twice that of the WT, and control the expression of 357 genes involved in diverse metabolic functions, including extracellular electron transfer and exopolysaccharide synthesis (Hernández-Eligio et al. 2025). Moreover, Δ*csrA* mutation improves the reduction of soluble Fe(III) using acetate as an electron donor, highlighting its role in pathways essential for biofilm development and electroactivity in highly efficient electroactive microorganisms. To further elucidate the mechanisms regulating biofilm formation and extracellular electron transfer in *G. sulfurreducens*, we investigated whether CsrA activity is modulated through interaction with the FliW protein.

### CsrA of *G. sulfurreducens* dimerizes

CsrA is known to form dimers in several bacteria. In *E. coli*, CsrA dimers bind to one or two sites on target mRNAs (Mercante et al. 2006; Yakhnin et al. 2011), and in *Pseudomonas aeruginosa*, the CsrA homolog RsmA also forms dimers that regulate target mRNAs (Chihara et al. 2021). To determine whether *G. sulfurreducens* CsrA forms dimers, we employed a LexA-based genetic system for analyzing protein homodimerization (Dimitrova et al. 1998; Daines and Silver 2000). In this system, the WT LexA DNA-binding domain (LexA_DBDwt_) is expressed from the pSR658 plasmid in the *E. coli* SU101 reporter strain, which carries a chromosomal *sulA*–*lacZ* transcriptional fusion containing a LexA_wt_ operator (Dimitrova et al. 1998; Daines and Silver 2000). LexA_DBDwt_ alone does not affect *sulA*–*lacZ* expression; however, when fused to a protein capable of dimerization, an active LexA complex is formed that binds the LexA_wt_ operator and represses *sulA*–*lacZ* transcription. To assess CsrA dimerization, we constructed the pSR658-*csrA* plasmid expressing a LexA_DBDwt_-CsrA fusion protein. The plasmid pSR658-HilD1, expressing LexA_DBDwt_-HilD, was used as a positive control, as dimerization of the LexA_DBDwt_-HilD fusion has been previously demonstrated (Paredes-Amaya et al. 2018). The empty pSR658 plasmid and the SU101 strain lacking any plasmid were used as negative controls. As shown in Fig. 1A, *sulA–lacZ* expression was detected in the SU101 and SU101/pSR658 strains, whereas expression was markedly reduced in the SU101/pSR658-HilD1 and SU101/pSR658-csrA strains expressing LexA_DBDwt_-HilD and LexA_DBDwt_-CsrA, respectively. Our results revealed that CsrA from *G. sulfurreducens* forms homodimers and interacts with FliW, which is consistent with mechanisms described in *B. subtilis*, *C. jejuni*, and *C. difficile* (Mukherjee et al. 2016; Bogacz et al. 2021; Zhu et al. 2021, 2025). Furthermore, in line with previous reports (Brutinel et al. 2010; Paredes-Amaya et al. 2018), we did not detect LexA_DBD_ expression in our Western blot analysis.

### FliW interacts with CsrA

In *B. subtilis*, *C. jejuni*, and *C. difficile* R20291, FliW antagonizes CsrA activity through direct protein–protein interaction (Mukherjee et al. 2016; Bogacz et al. 2021; Zhu et al. 2021, 2025). In *G. sulfurreducens*, the *fliW* gene is located immediately downstream of *csrA*, and both genes are co-transcribed as part of the *flgJKLMN–csrA–fliW* operon (Hernández-Eligio et al. 2025), suggesting a conserved regulatory relationship. To determine whether FliW modulates CsrA activity in *G. sulfurreducens*, we analyzed the interaction between these proteins using a LexA-based heterodimerization system (Dimitrova et al. 1998; Daines and Silver 2000).

This system relies on two variants of the LexA DNA-binding domain [wild-type (LexA_DBDwt_) and mutant (LexA_DBDmut_)] expressed from separate plasmids in the *E. coli* SU202 reporter strain, which carries a *sulA*–*lacZ* fusion controlled by a LexA hybrid operator. Expression of LexA_DBDwt_ or LexA_DBDmut_ alone does not repress *sulA*–*lacZ*. However, when fused to interacting proteins, heterodimer formation generates an active complex capable of binding the hybrid operator and repressing reporter expression. Using this approach, repression of *sulA*–*lacZ* was observed in strains co-expressing LexA_DBDwt_-CsrA with LexA_DBDmut_-FliW, or the reciprocal fusion combination, indicating direct interaction between CsrA and FliW. The positive heterodimerization control (LexA_DBDwt_-HilD and LexA_DBDmut_-HilE) behaved as expected (Paredes-Amaya et al. 2018), while all negative controls retained *sulA*–*lacZ* expression.

Western blot analyses confirmed expression of the corresponding fusion proteins, including LexA_DBDwt_-CsrA, LexA_DBDmut_-CsrA, LexA_DBDwt_-FliW, and LexA_DBDmut_-FliW. As observed in the homodimerization assays, LexA_DBD_ alone was not detected, consistent with previous reports (Brutinel et al. 2010; Paredes-Amaya et al. 2018). Together, these results support the conclusion that CsrA and FliW form heterodimers in *G. sulfurreducens*.

The interaction between CsrA and FliW was further validated by pull-down assays using purified CsrA–6His as bait. LexA_DBDwt_-FliW, but not LexA_DBDwt_ or LexA_DBDwt_-HilD, was specifically retained on the CsrA–6His-bound resin and detected by western blotting. This independent biochemical evidence confirms a direct and specific interaction between CsrA and FliW. Taken together, the LexA-based heterodimerization assays and pull-down experiments demonstrate that FliW directly interacts with CsrA in *G. sulfurreducens*, supporting a conserved regulatory mechanism in which FliW antagonizes CsrA activity through protein–protein interaction.

### The N55 residue in *G. sulfurreducens* CsrA is conserved and is involved in the interaction with FliW

The N55 residue is invariant across diverse bacterial species that contain CsrA-FliW regulatory systems (Murkherjee et al. 2016, Oshiro et al. 2021). In *B. subtilis*, the conserved N55 residue of CsrA has been shown to be critical for its interaction with FliW (Oshiro et al. 2021). Similarly, deletion of the two α-helices located at the C-terminal region of CsrA was reported to abolish binding to FliW in *G. thermodenitrificans* (Altagoer et al. 2016). In *G. thermodenitrificans*, the conserved N55 residue is located within the first α-helix of CsrA. An in silico structural analysis of the *G. sulfurreducens* CsrA protein using the AlphaFold Protein Structure Database (Varadi et al. 2024) predicts the presence of three β-strands in the N-terminal region followed by two α-helices in the C-terminal region (Supplementary Fig. 7). Consistent with observations in *B. subtilis* and *G. thermodenitrificans*, the *G. sulfurreducens* CsrA also shows a conserved N55 residue positioned within the first α-helix. In the present study, the observed restoration of *sulA*–*lacZ* expression in the SU202/pSR658-fliW + pSR659-csrA_N55D_ strain and the fusion proteins were detected by Western blot, demonstrating their correct expression. This observation leads us to suggest that the conserved N55 residue of CsrA has an important role in FliW-CsrA interaction in *G. sulfurreducens* as described in other bacteria.

### FliW is not required for soluble Fe(III) reduction in *G. sulfurreducens*

To investigate the role of FliW in growth, Fe(III) reduction, and biofilm development in *G. sulfurreducens*, a Δ*fliW* mutant was constructed using the scarless gene deletion system (Chan et al. 2015). Deletion of *fliW* did not affect the growth of *G. sulfurreducens* when acetate and fumarate were used as the electron donor and acceptor, respectively. Likewise, the reduction of soluble Fe(III) was not altered in the Δ*fliW* strain compared to the WT. These results indicate that FliW is not required for growth or for the reduction of soluble Fe(III) in *G. sulfurreducens*.

### FliW affects the expression of selected genes regulated by CsrA

A previous transcriptomic analysis of *G. sulfurreducens* biofilms revealed that deletion of *csrA* affects the abundance of mRNAs of 244 genes, including *gsu3268*, *gsu3274*, *gsu2236*, *gsu0972*, *gsu1944*, *gsu3014*, *gsu0597*, and *gsu0018* (Hernández-Eligio et al. 2025). In the present study, *gsu3268*, *gsu3274*, and *gsu0972* exhibited lower transcript levels in the Δ*fliW* strain but increased levels in the Δ*csrA* strain. Conversely, *gsu1944*, *gsu3014*, and *gsu0597* were upregulated in the Δ*fliW* strain but downregulated in Δ*csrA*. The opposite expression patterns suggest that FliW influences the CsrA-dependent regulation of these transcripts Intriguingly, the expression of *gsu2236* and *gsu0018* followed the same trend in both the Δ*fliW* and Δ*csrA* strains, with *gsu2236* being upregulated and *gsu0018* downregulated. This observation suggests that additional regulatory mechanisms, including global physiological adjustments, may influence the expression of these genes independently of the FliW–CsrA interaction.

### FliW negatively affects biofilm formation in *G. sulfurreducens*

In *G. sulfurreducens*, CsrA plays a central role in regulating biofilm formation and extracellular electron transfer in MFCs (Hernández-Eligio et al. 2025). Given the interaction between FliW and CsrA, we investigated whether FliW influences biofilm development in this organism. Previous studies have shown that *G. sulfurreducens* readily forms biofilms on FTO electrodes, a material that promotes extensive surface coverage and supports the formation of highly electroactive biofilms due to its conductive properties (Huerta-Miranda et al. 2019; Rodríguez-Torres et al. 2024). The reduction in biofilm thickness and cell viability observed in the Δ*fliW* strain suggests that, in the absence of FliW, CsrA is unrestrained and can regulate its target RNAs involved in biofilm formation. However, the specific target RNAs that mediate this effect have not yet been identified and will be investigated in future studies.

### Characterization of *G. sulfurreducens* electroactive biofilms

According to electrochemical characterization, the *ΔcsrA* mutants stand out in terms of thermodynamic and kinetic behavior compared with WT and *ΔfliW* biofilms. A slight negative shift in OCP values was observed upon NaAc addition indicating a metabolic response to substrate oxidation (Risso et al. 2008; Hernández-Eligio et al. 2022; Rodríguez-Torres et al. 2024). The sigmoidal voltammograms on FTO electrodes exhibited by all three strains, indicates electroactivity and extracellular electron transfer, were consistent with previous reports for *G. sulfurreducens* on this support material (Hernández-Eligio et al. 2022; Huerta-Miranda et al. 2023).

Δ*csrA* biofilms were characterized by a more negative OCP and a more positive E0′ relative to WT and Δ*fliW* biofilms, indicating a change in their extracellular electron transfer capabilities, which may be associated with the resulting phenotype produced by the mutation.

This observation appears consistent with patterns noted in other electrogenic mutants, such as Δ*gsu1771* (Hernández-Eligio et al. 2022), although Δ*gsu1771* displayed a pronounced − 100 mV shift in response to acetate, a sensitivity not detected in the present study.

The observed *i*_*L*_ values demonstrated that Δ*csrA* biofilms reached higher currents both in the presence and absence of acetate, reaching levels approximately 13-fold greater than the wild-type DL1 strain. This pronounced performance gap, larger than that observed in a previous MFC experiment (1.5-fold), underscores the fundamental differences between experimental systems (Hernández-Eligio et al. 2025). In three-electrode configurations, the measured current reflects the intrinsic activity of the anode biofilm under controlled potentiostatic conditions, whereas MFC currents are constrained by the cathode reaction and overall system design (Malvankar et al. 2012). Thus, three-electrode setups provide a more reliable means of assessing biofilm electroactivity, enabling better comparison of the overall functional impact of genetic modifications (Beyenal and Babauta 2015; Marsili et al. 2010).

The electrochemical activity of Δ*csrA* biofilms, as revealed by CV and SWV, is consistent with other observed phenotypes such as the biofilm’s thickness and cell viability. In contrast, Δ*fliW* biofilms resemble the WT in electrochemical behavior, suggesting that Δ*fliW* biofilms are not highly affected in their electron transfer capabilities. Differences in redox peaks and currents highlight the impact of CsrA and FliW on the extracellular electron transfer network in *G. sulfurreducens* (Hernández-Eligio et al. 2022; Rodríguez-Torres et al. 2024; Howley et al. 2023).

### Regulation model of CsrA/FliW in *G. sulfurreducens*

CsrA (RsmA) is a post-transcriptional regulator that controls numerous essential cellular processes in bacteria. Typically, CsrA activity is regulated by sRNAs of the CsrB/CsrC (RsmY/RsmX) family, which contain multiple binding sites for CsrA. However, in bacteria such as *B. subtilis*, *C. jejuni*, *C. difficile*, and *G. thermodenitrificans*, where these sRNAs are absent, the FliW protein has been found to interact directly with CsrA (Altagoer et al. 2016; Mukherjee et al. 2016; Oshiro et al. 2021; Bogacz et al. 2021; Zhu et al. 2025). CsrA in these bacteria contains a C-terminal extension absent in homologs regulated by sRNAs. This extension includes residues critical for FliW binding, with the conserved asparagine N55 proposed as a key amino acid mediating the interaction (Oshiro et al. 2021). Similarly, in *G. sulfurreducens*, no CsrB/C-family sRNAs have been characterized, but the *fliW* gene is located downstream of *csrA*, and CsrA possesses an extended C-terminal region with the conserved N55 residue.

**Fig. 8:**
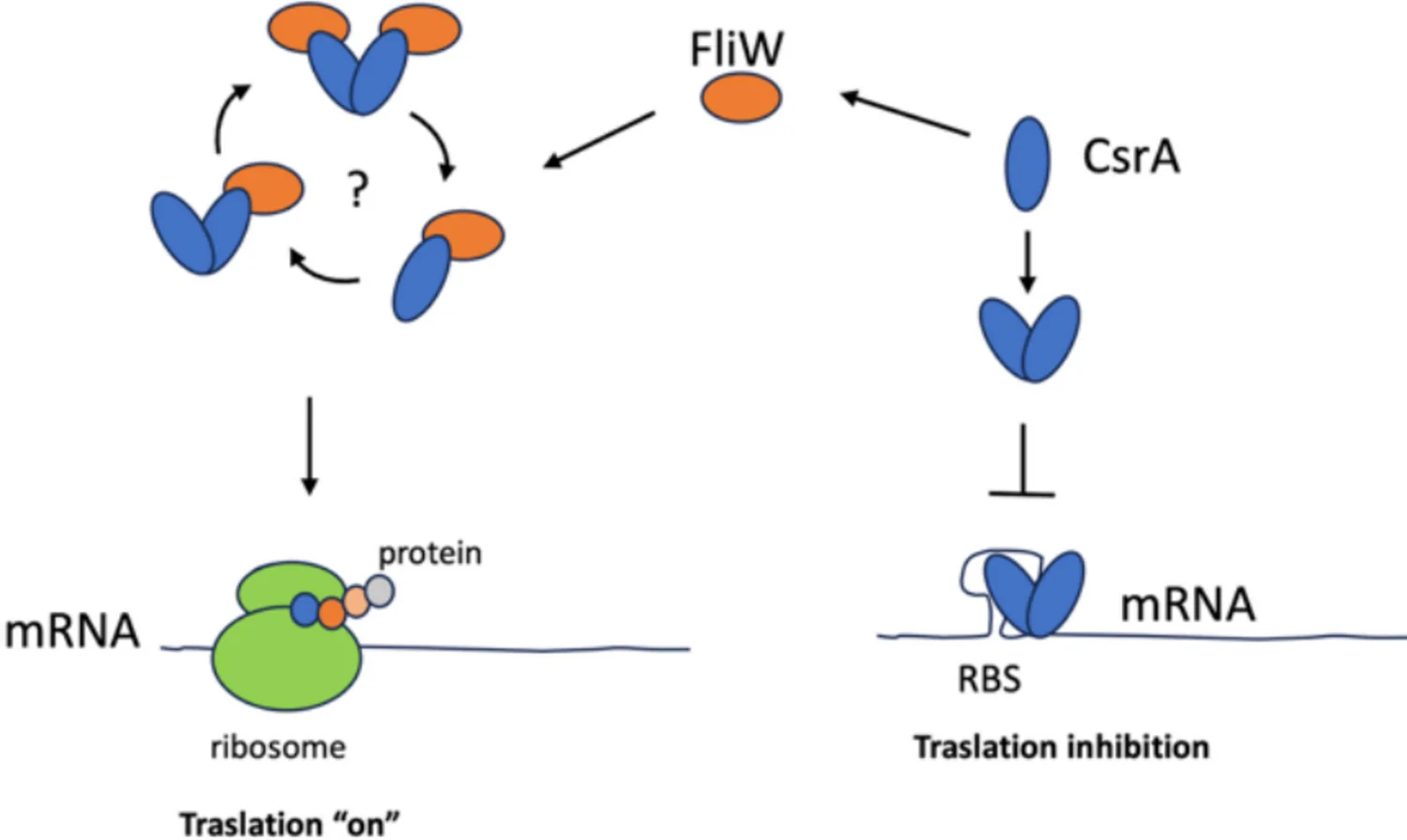
Proposed working model for the CsrA/FliW interaction in *G. sulfurreducens*. Under the proposed hypothesis, the binding of FliW to CsrA antagonizes CsrA activity, thereby modulating the transcript abundance of genes associated with biofilm formation and extracellular electron transfer

Based on our results and previous studies, we propose a working hypothesis in which FliW interacts directly with CsrA to modulate the transcriptomic profile involved in electroconductive biofilm formation in *G. sulfurreducens* (Fig. 8). In this proposed model, CsrA is predicted to positively or negatively control the translation of specific transcripts, while FliW counteracts this activity through direct binding. This interaction is mediated by the C-terminal region of CsrA and specifically involves the conserved N55 residue, consistent with mechanisms described in other bacteria lacking CsrB/C-family sRNAs (Oshiro et al. 2021; Zhu et al. 2025). This putative post-transcriptional regulatory strategy suggests a functional link between protein–protein interaction and the control of biofilm development and extracellular electron transfer. However, it is important to note that although our phenotypic and expression data support this model, the direct binding of CsrA to the proposed mRNA targets remains to be experimentally validated. Furthermore, additional studies are required to determine whether other factors, such as FliC, participate in this regulatory network, as reported in other bacterial systems (Zhu et al. 2025).

## Conclusion

For the first time, this study describes the identification of the CsrA-FliW interaction and proposes a putative regulatory mechanism in *G. sulfurreducens,* linking post-transcriptional regulation of the biofilm formation and structure, and the effect on the electron transfer to the final electron acceptors, resulting in an altered electrochemical performance. This regulatory network expands our understanding of genetic regulation in electroactive bacteria and can serve as a basis for optimization strategies of MFCs and biofilm-based bioremediation systems (Hernández-Eligio et al. 2025; Huerta-Miranda et al. 2019; Rodríguez-Torres et al. 2024).

## Supplementary Information

Below is the link to the electronic supplementary material.

Supplementary file1 (PDF 2340 kb)

## Data Availability

No datasets were generated or analysed during the current study.
