# Supplementary material for: FliW regulates biofilm formation in Geobacter sulfurreducens through interaction with CsrA
Source: Appl Microbiol Biotechnol. 2026 May 28;110(1):223. doi: 10.1007/s00253-026-13884-0 (PMC13407953; doi:10.1007/s00253-026-13884-0)
Supplement: Supplementary file 1 — Supplementary file1 (PDF 2340 kb) [file 253_2026_13884_MOESM1_ESM.pdf]

SUPPLEMENTARY INFORMATION

FliW regulates biofilm formation *in Geobacter sulfurreducens* through interaction with CsrA

Jessica Cholula-Calixto,[1] Guillermo Huerta-Miranda,[1] Bernardo Jaramillo-Rodríguez, [1] Víctor H. Bustamante,[1] Katy Juárez,[1*] Alberto Hernández-Eligio[1,2*]

[1]Departamento de Microbiología Molecular, Instituto de Biotecnología, Universidad Nacional Autónoma de México, Av. Universidad 2001, Col. Chamilpa CP 62210, Cuernavaca Morelos, México.

[2]Investigador por México, Secretaría de Ciencia, Humanidades, Tecnología e Innovación, Av. Insurgentes Sur 1528, Col. Crédito Constructor CP 03940, Ciudad de México, México.

Corresponding author

AH-E correspondence to alberto.hernandez@ibt.unam.mx

KJ correspondence to katy.juarez@ibt.unam.mx

ORCID

AH-E 0000-0002-2787-8732

KJ 0000-0002-4400-4674

GH-M 0000-0003-1183-3668

VB 0000-0002-8852-150X

BJ-R 0000-0002-4081-7440

JC-C 0009-0004-5923-7990

Keywords: CsrA posttranscriptional regulator, biofilm, *Geobacter sulfurreducens*, electrochemical, LexA-based genetic system

Supplementary Table 1 List of strains, plasmid, and oligonucleotides used in this work

Strain, plasmid or oligonucleotide	Description	Reference
Bacteria		
Geobacer sulfurreducens		
DL1	Wild type strain	Caccavo et al. 1994
Δ*fliW*	DL1 with fliW deletion	This work
Δ*csrA*	DL1 with *csrA* deletion	Hernández-Eligio et al. 2025
Eschericha coli		
DH5α	Laboratory strain	Invitrogen
S17-1	*recA pro hsdR RP*-4-2-*Tc*::*Mu*-*Km*::*Tn*7	Simon et al. 1983
SU101	Reporter strain for homodimerizarion assay, Kan^R	Dmitrova et al. 1998
SU202	Reporter strain for heterodimerizarion assay, Kan^R	Dmitrova et al. 1998
BL21	*fhuA2 [lon] ompT gal [dcm] ΔhsdS*	New England Biolabs
Plasmids		
pK18mobsacB	Plasmid suicide, for scarless deletions	Simon el at. 1983
pJCC	pK18mobsacB with the flanking regions of *fliW* gene	This work
pJET1.2	Subcloning plasmid, Ap^R	Thermo
pJET-RRflg	pJET1.2 with the regulatory region of the *flgJ* gene (RRflg) fragment	Hernández-Eligio et al. 2025
pJET-RRflg-fliW	pJET-RRflg with *fliW* gene	This work
pRG5.1	Bacterial expression vector, Sp^R	Kim et al. 2005
pRG5.1-RRflg-fliW	pRG5.1 with the fusion RRflg-fliW	This work
pSR658	Plasmid expressing the $LexA_{DBDwt}$ for homodimerization assay, Tc^R	Daines et al. 2000
pSR658-HilD1	pSR658 derivated expressing the $LexA_{DBDwt}$-HilD, Tc^R	Paredes-Amaya et al. 2019
pSR658-fliW	pSR658 derivated expressing the $LexA_{DBDwt}$-FliW, Tc^R	This work
pSR658-csrA	pSR658 derivated expressing the $LexA_{DBDwt}$-CsrA, Tc^R	This work
pSR658-csrAN55D	pSR658-csrA derivated expressing the $LexA_{DBDwt}$-$CsrA_{N55D}$, Tc^R	This work
pSR659	Plasmid expressing the $LexA_{DBDmut}$ for heterodimerization assay, Ap^R	Daines et al. 2000
pSR659-HilE1	pSR659 derivated expressing the $LexA_{DBDmut}$-HilE, Ap^R	Paredes-Amaya et al. 2019
pSR659-fliW	pSR659 derivated expressing the $LexA_{DBDmut}$-FliW, Ap^R	This work
pSR659-csrA	pSR659 derivated expressing the $LexA_{DBDmut}$-CsrA, Ap^R	This work

pSR659-csrAN55D	pSR659-csrA derivated expressing the $LexA_{DBDmut}$-$CsrA_{N55D}$, Ap^R	This work
pET24a	Plasmid for polyhistidine fusion protein expression	Novagen
pET24a-csrA	pET24a derivated expressing CsrA-6hist fusion protein	This work
Oligonucleotides		
for Δ*fliW* mutant strain		
1Fw	AATTGAATTCCGGGAGAAGTAGGTGCGG	This work
2Rev	GCGGACGGGAAGGAGTAATACGGCGTGATCTGGTAACCA	This work
3Fw	TGGTTACCAGATCACGCCGTATTACTCCTTCCCGTCCGC	This work
4Rev	AATTCTGCAGGATGTCATCCTCGGGCTC	This work
for CsrA expression		
pETCsrAfw	AATTCATATGTTAGTACTGACCAG	This work
pETCsrARev	AATTCTCGAGCTCCTTCCCGTCCG	This work
for homodimerization and heterodimerizarion assay		
FwcsrA	AATTCTCGAGTTAGTACTGACCAG	This work
RvcsrA	AATTGGTACCTTACTCCTTCCCGTCCG	This work
FwfliW	AATTCTCGAGGTGAACGTGACCAC	This work
RvfliW	AATTGGTACCTCACGCCGCCGTAA	This work
csrAN55Dfw	GTTCAGCGCGAA**GAC**CAGATTGCCGCTTCG	This work
csrAN55DRv	CGAAGCGGCAATCTG**GTC**TTCGCGCAGAAC	This work
for ΔfliW complementation		
NdeIfliWfw	ACGCTCATATGGTGAACGTGACCACCACG	This work
HindfliwRv	AGCTCAAGCTTTCACGCCGCCGTAACCTC	This work

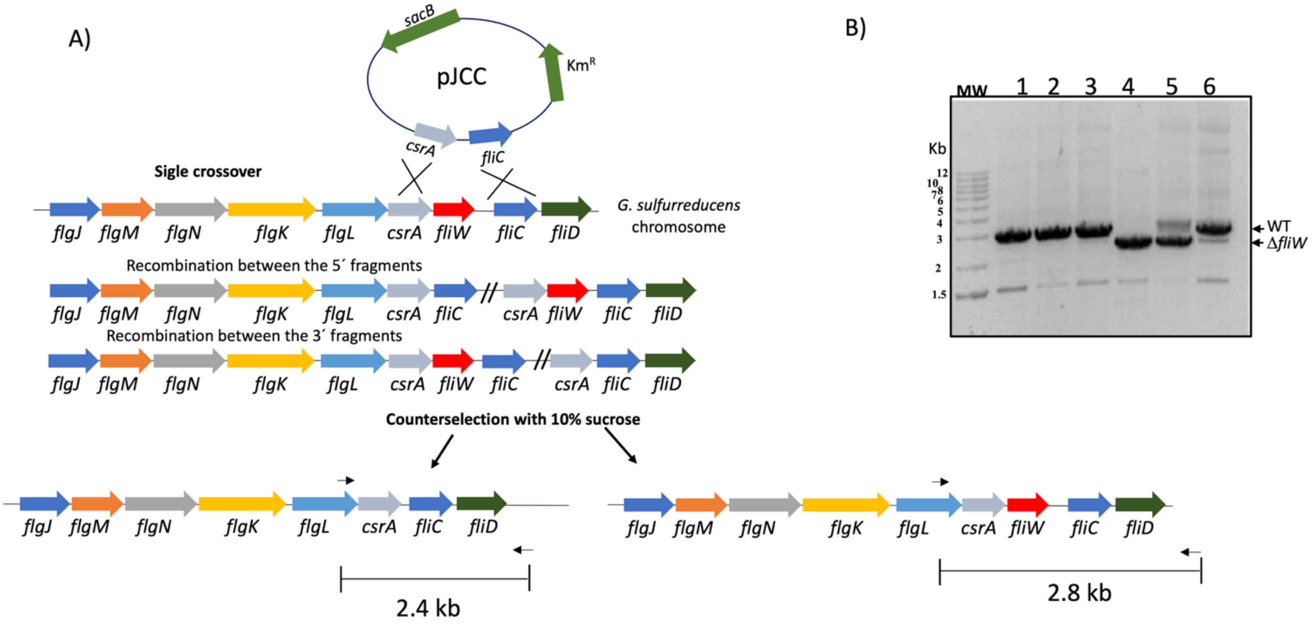

Supplementary Fig. 1 Construction of *G. sulfurreducens* Δ*fliW* mutant strain. A) Diagram of construction of *G. sulfurreducens* Δ*fliW* mutant. Arrows showed the oligonucleotide pair used and expected sizes. B) Agarose gel with the PCR fragments of *fliW* locus. Line 1 shows the fragment amplified using DNA from wild type strain, lines 2-6 shows the fragments obtained using DNA from candidate colonies of Δ*fliW* mutants. Only the candidate 3 amplifies the *fliW* mutant locus (line 4). 1 Kb Plus DNA ladder was used as molecular weight (Biolabs). Arrows indicate expected molecular weights.

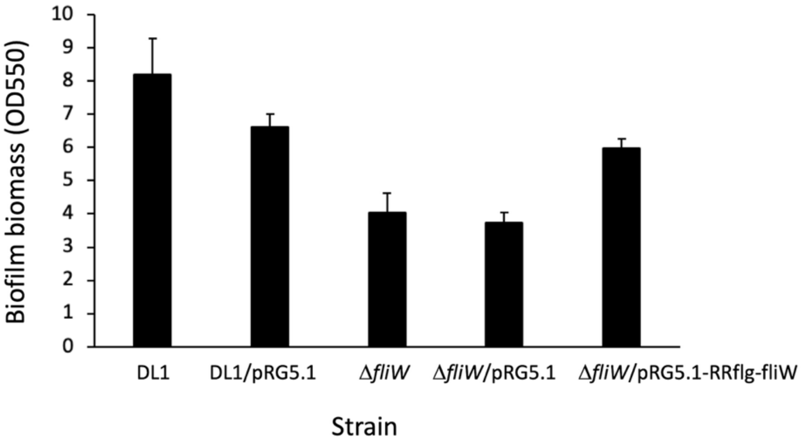

Supplementary Fig. 2 Average biomass of *G. sulfurreducens* biofilms. DL1 (wild-type), DL1/pRG5.1 (wild-type with empty vector), Δ*fliW* (mutant strain), Δ*fliW* (mutant strain with empty vector), and Δ*fliW*/pRG5.1-RRflg-fliW (mutant strain with *fliW* gene). Biofilm exopolysaccharide matrix generation was estimated using the crystal violet method (Merritt et al. 2005). The cells were incubated in acetate-fumarate for 72 h at 25°C, after which the optical density at 600 nm (OD_{600}) of the culture was measured before discarding the culture broth and staining the biofilms for 20 min with 100 µl of a 0.1 % (w/v) aqueous solution of crystal violet. After staining, the biofilms were washed with Milli-Q H_2O and dried for 1 h at 37°C. The biofilm-associated crystal violet was then re-solubilized with 30% (v/v) acetic acid. The biofilm biomass was then estimated based on the OD of the solution at 550 nm and the average biofilm biomass for each strain was obtained from four replicate samples. Biofilm growth was reported as the biofilm biomass stained with crystal violet (OD_{550}) relative to the OD_{600} of the planktonic culture.

A)

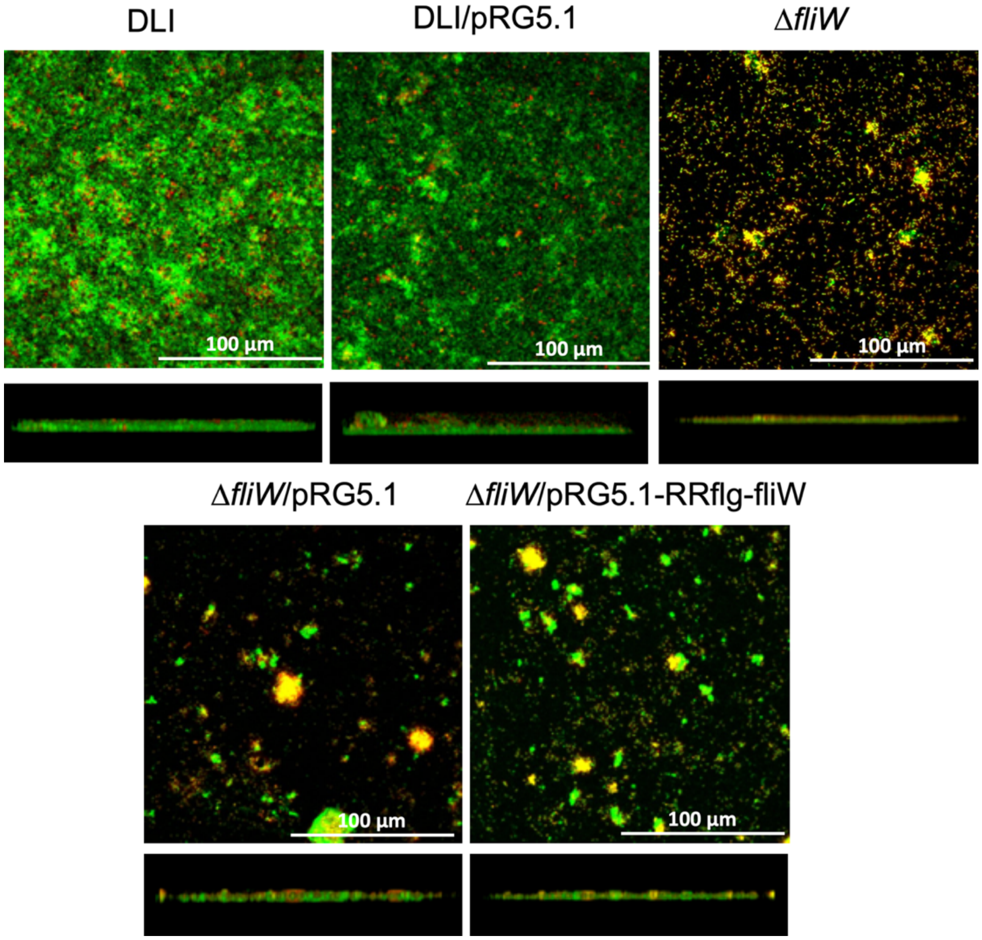

B)

	Thickness µm	% viability(live cells)
DL1	17.51 ±3.21	77.47 ±4.36
DL1/pRG5.1	22.3 ±1.974	68.58 ±0.974
Δ*fliW*	8.69 ±0.577	56.2 ±1.35
Δ*fliW*/pRG5.1	11.23 ±1.34	58.87 ±1.326
Δ*fliW*/pRG5.1-RRflg-fliW	14.4 ±3.42	81.33 ±2.457

Supplementary Fig. 3 Complementation of Δ*fliW* mutant strain. A) CLSM images of DL1 (WT), DL1/pRG5.1 (empty vector), Δ*fliW*, Δ*fliW*/pRG5.1 (empty vector), and Δ*fliW*/pRG5.1-RRflg-fliW biofilms grown on FTO electrodes in acetate-fumarate medium at 72 h. The top and bottom panels show top and side view projections of *G. sulfurreducens* strains biofilms at 72 h. B) Thickness (µm) and % of viability of *G. sulfurreducens* biofilms grown on FTO electrodes in acetate-fumarate medium at 72 h.

The Box Plot in Supplementary Fig. 4A shows the distribution and range of potentials recorded for each strain and condition analyzed in this work. The OCP values were analyzed using one-way analysis of variance (ANOVA) followed by Tukey's multiple comparison test. A p-value of < 0.05 was considered statistically significant. All analyses were performed using Prism 10 (version 10.5.0; GraphPad Software, LLC). In the Box Plot, the Compact Letter Display (CLD) indicates that samples that share no letters are significantly different (e.g., FTO has the letter A and differs from all the others). This is the best way to see similarities. For example, the *ΔcsrA*-48h sample has the letters f and g, and the *ΔfliW*-72h sample has the letter g, indicating no significant difference between them. Supplementary Fig. 34 shows the 95% Confidence Interval Chart (Tukey). This chart plots the difference between the group means. If the confidence interval for a comparison crosses the zero line, it means there is no statistically significant difference between those two groups. If the interval is far from zero, the difference is significant.

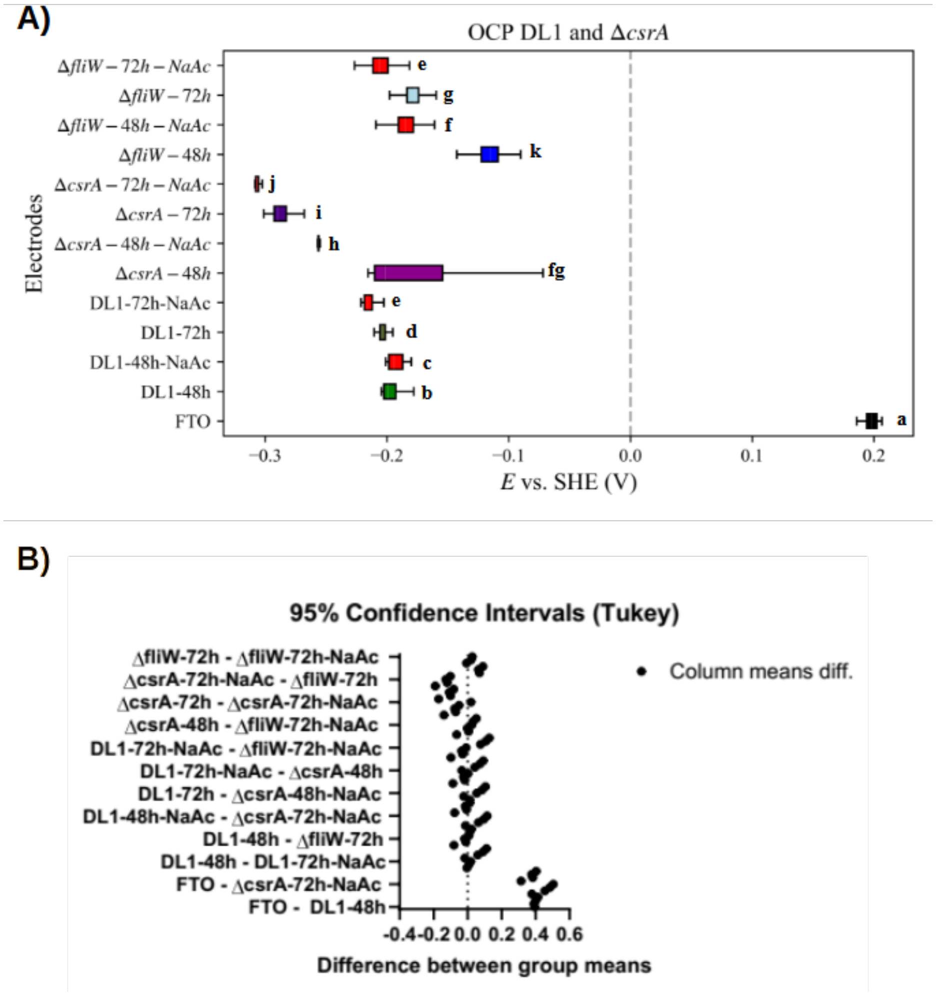

Supplementary Fig. 4 A) Open circuit potential (OCP) distribution of wild type (DL1), *ΔcsrA,* and *ΔfliW* biofilms on FTO at 48 and 72 h of biofilm development, both without and in the presence of NaAc. The boxes represent the statistical distribution of 95% of the data (N = 2). The letters represent the CLD system. Means not sharing a letter are significantly different by the Tukey test at the 5% level of significance. B) 95% Confidence Interval Chart (Tukey): this graph plots the difference between the means of the groups.

The Nernst-Monod model operates on key assumptions: a uniformly conductive biofilm matrix, exclusive electron transfer from active biomass to this matrix, a reaction rate dependent on local substrate concentration and potential, negligible charge-transfer resistance and ionic limitations, stable pH, and rapid, reversible electron transfer from cells to the matrix.

$$i = i_L \frac{S}{K_S + S} \left[\frac{1}{exp\left(-\frac{\alpha n F (E - E_{ka})}{RT}\right)} \right] \qquad \text{(Eq. 1)}$$

Supplementary Fig. 5 The Nernst-Monod equation (Eq. 1) is a hybrid of the Monod and Butler-Volmer equations, parameterized for bioelectrochemical systems: where i is the steady-state current, i_L is the maximum current, S is the substrate concentration (0.011 mol/L, measured in terms of COD at 48 h in the WT NBAF medium; Dhar et al. 2016), K_S is the half-saturation constant (1.2×10^{-4} mol/L), α is the charge transfer coefficient, E is the electrode potential, E_{ka} is the formal potential, R is the gas constant, T is the temperature, n is the number of electrons, and F is the Faraday constant.

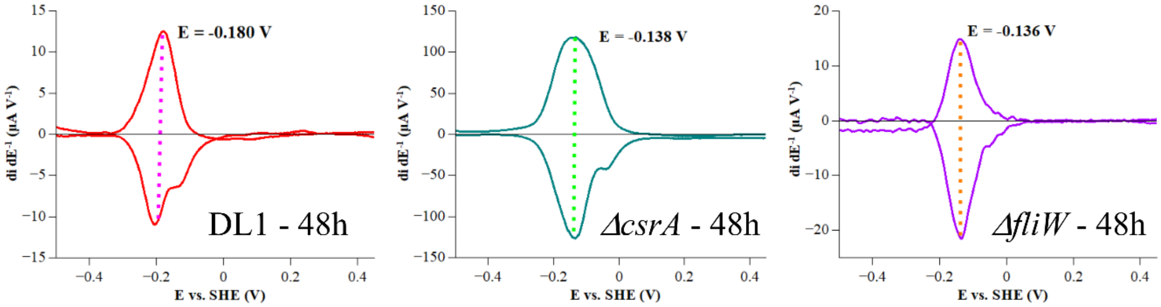

Supplementary Fig. 6 First derivative of the voltammetric responses at 1 mV/s scan rate obtained in MB medium in the absence of sodium acetate. The mid-point potential is signaled by the dotted-lines.

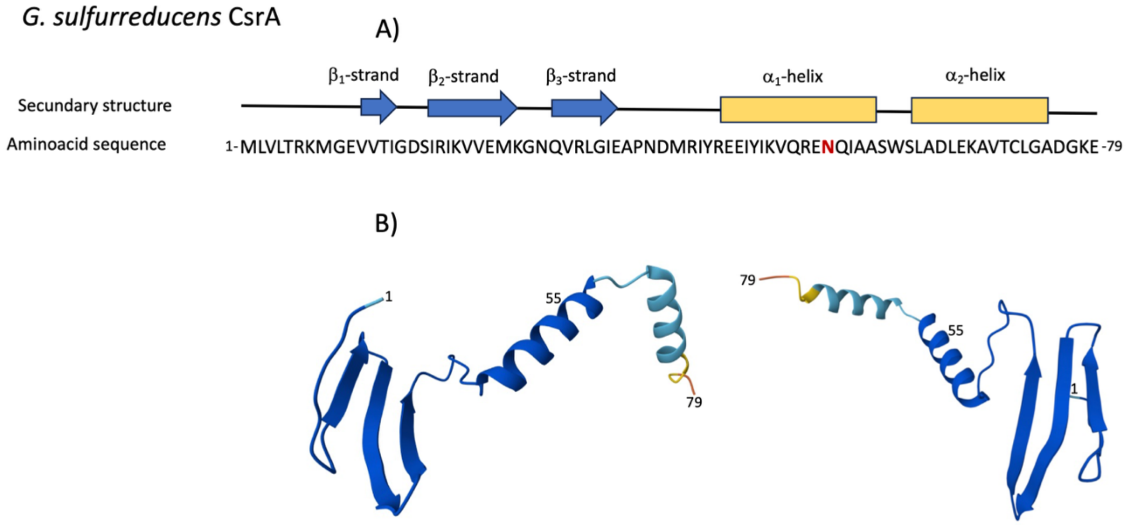

Supplementary Fig. 7 Structure of the *G. sulfurreducens* CsrA protein. A) The secondary structure elements of CsrA are labeled and shown in blue (β-strand) and yellow (α-helix). The amino acid sequence is shown, with asparagine 55 (N) highlighted in red. B) The overall structure of CsrA is depicted. The structure of two CsrA monomers is predicted using AlphaFold. Numbers 1 and 79 show the N- and C-terminal, respectively. Asparagine 55 (N55) is shown in the α_1-helix

References

Caccavo FJR, Lonergan DJ, Lovley DR, Davis M, Stolz JF, Mcinerney MJ (1994) *Geobacter sulfurreducens* sp. nov., a Hydrogen- and Acetate- oxidizing dissimilatory metal-reducing microorganism. *Appl Environ Microbiol* 3752-37589. doi:10.1128/aem.60.10.3752-3759.1994

Daines DA, Silver RP (2000) Evidence for multimerization of neu proteins involved in polysialic acid synthesis in *Escherichia coli* K1 using improved LexA-based vectors. *J Bacteriol* 182: 5267-5270. doi:10.1128/jb.182.18.5267-5270.2000

Dmitrova M, Younes-Cauet G, Oertel-Buchheit P, Porte D, Schnarr M, Granger-Schnarr M (1998) A new LexA-based genetic system for monitoring and analyzing protein heterodimerization in *Escherichia coli*. *Mol Gen Genet* 257:205-212. doi:10.1007/s004380050640

Hernández-Eligio A, Vega-Alvarado L, Liu X, Cholula-Calixto J, Huerta-Miranda G, Juárez K (2025) The role of CsrA in controls the extracellular electron transfer and biofilm production in *Geobacter sulfurreducens*. *Front Microbiol* 16:1534446. doi:10.3389/fmicb.2025.1534446

Kim B-C, Leang C, Ding Y-H, Glaven R H, Coppi M V, Lovley DR (2005) OmcF, a putative c-type monoheme outer membrane cytochrome required for the expression of other outer membrane cytochromes in *Geobacter sulfurreducens*. *J Bacteriol* 187:4505–4513. doi:10.1128/JB.187.13.4505-4513.2005

Merritt JH, Kadouri DE, O’Toole GA (2005) Growing and analyzing static biofilms. *Curr Protoc Microbiol* 00: 1B.1.1-1B.1.17. doi:10.1002/9780471729259.mc01b01s00

Paredes-Amaya CC, Valdés-García G, Juárez-González VR, Rudiño-Piñera E, Bustamante V (2018) The Hcp-like protein HilE inhibits homodimerization and DNA binding of the virulence-associated transcriptional regulator HilD in *Salmonella*. *J Biol Chem* 293(17):6578-6592. doi:10.1074/jbc.RA117.001421

Simon R, Priefer U, Pühler A (1983) A broad host range mobilization system for in vivo genetic engineering: transposon mutagenesis in gram negative bacteria. *Nat Biotech* 1:784–791. doi:10.1038/nbt1183-784
